# Religiosity, neutrality, fairness, skepticism, and societal tranquility: A data science analysis of the World Values Survey

**DOI:** 10.1371/journal.pone.0245231

**Published:** 2021-01-11

**Authors:** Leigh Allison, Chun Wang, Jessica Kaminsky

**Affiliations:** 1 Department of Civil and Environmental Engineering, University of Washington, Seattle, Washington, United States of America; 2 College of Education, University of Washington, Seattle, Washington, United States of America; Sun Yat-sen University, CHINA

## Abstract

Quantitative models of social differences have not only made major contributions to the fields of cross-cultural anthropology, psychology and sociology, but also have allowed for interdisciplinary studies that bring together engineering, life sciences, and social sciences. In this study, the authors use a data science approach to discover a set of quantitative social dimensions based on the World Values Survey, a nationally representative survey covering 60 countries and 90,000 individuals. Five national social dimensions, representing 198 questions and 56 countries are discovered using multidimensional item response theory (MIRT). They are (1) Religiosity, (2) Neutrality, (3) Fairness, (4) Skepticism, and (5) Societal Tranquility. This approach is unique from previous quantitative models because it groups responses by country and analyzes binary, nominal, and ordinal survey questions. It is possible today due to recent advancements in computing power and programming. Furthermore, this methodology tests the validity of previous quantitative dimensions and finds that some of the existing social and cultural dimensions are not clearly discernable. Therefore, this model provides not only more a rigorous methodology but also new social dimensions which more accurately quantify underlying differences across countries in the World Values Survey. Like other quantitative cross-cultural models, this model is a deeply simplified representation of national social differences. However, it is a useful tool for modeling national differences and can be used to help us understand the impacts of social preferences and values on different political, economic, and development variables.

## Introduction

Social values impact decisions and views of people and organizations across the world from how governments are organized to how people treat one another. Because of this, scholars have created quantitative variables to represent them for research and business applications. As quantitative variables, social values can be estimated, predicted, and compared across different groups of people [1]. Of course, social values cannot be measured directly by a single question. As such, social values are latent variables that must be measured through a series of manifest variables. For example, in a well-known quantitative model of culture, Hofstede identified four social values that he claims are cultural dimensions. One of these is individualism versus collectivism; it is based on a series of question responses (e.g. importance of family time, good working and living conditions, and job security) which have been aggregated into a series of national scores for individualism [2]. Hofstede and other scholars with similar approaches have been celebrated for creating tools for national comparison of social values, but also criticized for limitations in sample size, question choice, and reproducibility of data analysis. In this article, we present a new approach for measuring social values at the national level which more accurately measures and represents empirically observed quantitative differences.

There are five differences from existing models that in combination make this model unique. First, this model uses nationally representative data from the World Values Survey (WVS). The WVS has been distributed in seven waves over the last 37 years to nationally representative populations worldwide [3]. The dataset and final dimensions include nine countries from northern Africa, the Middle East, and eastern Europe, which are rarely represented in existing models. Second, the questions used to create the model are not preselected based on existing cultural theories or variability over time; instead multivariate statistical modeling allowed the dimensions to emerge from all 198 questions in the dataset. Third, this model accounts for the distribution of responses within a country rather than relying on country means. Fourth, this model is computationally reproducible. The analysis code is written in R, a free statistical programming software, and is available for download at https://github.com/Laalliso/SocialDimensions. By writing and providing the analysis in R, as opposed to instructions for manual calculations, our methodology is transparent, accessible, and automatically reproducible to future researchers. Fifth, this analysis method is adaptable to previous or future WVS data sets. All waves of the WVS data are publicly available for download; however, since the WVS questions can change with each wave, data cleaning would be required before applying the analysis code published with this study.

Therefore, this article begins a discussion of previous quantitative models all of which have provided insight as well as limitations in the study of cross-cultural values. Then we proceed into a detailed description of how the social dimensions were developed. Next, we describe our results showing not only that the discovered social dimensions measure meaningful values but also that they correlate with other national context variables. Finally, we recommend that future studies use these social dimensions to add to our current understandings of how values differ across societies and provide new perspectives for real social benefit.

## Existing cross-cultural & social dimension models

Cross-cultural scholars have taken a multitude of approaches to do describe how culture changes across different societies. In 1980, Hofstede introduced the idea of cultural dimensions which numerically quantified cultural differences between nations [4]. In 1994, Schwartz developed a questionnaire which measured ten individual values and seven cultural values [5]. In 1997, Inglehart was the first to use a nationally representative sample to document how inductively-discovered cultural dimensions change generationally [6]. In 2004, House et al. distributed the GLOBE survey and used a multilevel factor analysis to discover nine leadership values that vary significantly across countries [7]. Table 1 shows these four well-known empirical models. They all were developed with more than 20 countries, measure two or more values, and have subsequently been applied in other academic studies. For a more comprehensive list of existing cultural studies, please refer to Minkov’s 2013 book [8]. Please note that in 2010, Hofstede collaborated with Minkov to add two dimensions to his framework. Those dimensions were based on a combination of studies, including most prominently the World Values Survey [9]. Since the focus of the analysis was countries, they reported 93 countries for the two new dimensions, and the original four dimensions expanded 76 countries based on additional data collection using the Values Survey Module developed from the 1980 questions and subsequently updated over time [10]. An asterisk identifies these special conditions in Table 1.

**Table 1:** Existing cultural models.

Model		Number of Dimensions	Sample		Nationally Representative
Primary Author	Year		# of Countries	# of Respondents	
Hofstede	1980^a^	4	40	88,000	No
	2010^b^	6	93*	Varies*	No
Schwartz	1994^c^	7	38	12,900+	No
	2009^d^	7	72	55,022	No
Inglehart	1997^e^	2	43	60,000	Yes
	2000^f^	2	65	165,594	Yes
House	2004^g^	9	59	17,370	No

a—[2]

b—[9]

c—[5]

d—[11]

e—[6]

f—[12]

g—[7].

Over time, both Hofstede’s and Inglehart’s dimensions have been improved and criticized by colleagues; however, they have retained the support of the fundamental idea that culture can be quantitively measured in an insightful way. Most recently, Beugelsdijk and Welzel (2018) created three new dimensions by combining the ideas of Hofstede and Inglehart. They used nationally representative data to improve upon Hofstede’s dimensions and show how their new dimensions change over time [13].

In the majority of existing cultural models, values were measured using theoretically constructed questionnaires. For example, Schwartz and GLOBE developed questionnaires based on specific theoretical knowledge of the values. Schwartz proposed ten values that he believed all people hold at varying levels of importance. He developed a questionnaire that measured those values and distributed it to school teachers and students around the world [5, 14]. Schwartz and collaborators tested these ten values among individuals in many different cultures, but determined that only seven of the values are useful in measuring cultural differences across countries [15–17]. More recently, he repeated his study with a much wider sample of teachers and students and added additional clarification on the creation of the country scores [11, 18, 19]. While the majority of researchers accept the values that Schwartz has identified, there remains criticism of the broad definition of his values [8]. Interestingly in 2011, Fischer and Schwartz published a study that suggested that his questionnaire as well as a subset of the questions from Inglehart’s study only represent a small amount of the variability between countries [20].

The GLOBE study developed a questionnaire to understand leadership and organizational behavior by building on the dimensions that Hofstede identified in his study. A major difference in the GLOBE questionnaire was the addition of new concepts and how the questions were phrased [7]. An intense debate continues today about what was measured. Many scholars argue that instead of measuring culture as it is and should be, the study measured stereotypes and ideologies [8, 21, 22].

In his own words (1980), Hofstede used an “eclectic” combination of theoretical reasoning and statistics to develop his four cultural dimensions. For example, he developed the Uncertainty Avoidance Index (UAI) based around a question asking how often the employee felt nervous or tense at work. Theoretical reasoning guided him towards that primary question and data mining identified which other questions in the survey had correlations with it. For two of his other dimensions, he subset 14 questions from the original survey related to work goals and used a factor analysis to extract two latent factors. The first factor is referred to as the Individualism Index (IDV), and the second factor was identified as the Masculinity Index (MAS), based on a separate analysis on the effects of gender on work goals [2]. Due to his claims of broad generalizability, Hofstede has received considerable criticism about the measurement validity since the IBM questionnaire was developed to study employees’ opinions of a specific company [23–25]. In the years, since the first and second publications of *Culture’s Consequence*, Hofstede and collaborators have continued to compare his four dimensions to other data sets collected in ways that are more representative of a nation’s population. They argue that while the original model may have been based on less than ideal data, their dimensions of uncertainty avoidance, power distance, individualism, and masculinity are distinguishable [26]. Recently, however, Hofstede’s dimensions have been criticized and invalidated due to lack of internal stability and predictive properties in comparison to data from the World Values Survey and an Itim International survey [27, 28].

Inglehart took a different approach in 1997. Working with the second wave of the World Values Survey [29], Inglehart first constrained his analysis to questions that focused on modernist and postmodernist values. Using averages and other aggregate values of questions, he performed a principal component analysis resulting in two dimensions. He repeated analysis with the first (1980) and second (1990) waves of the WVS to confirm the factor structure with the 23 variables included in both surveys and that countries scored similarly over time [6]. Starting in 2000 and in the following publications of WVS data, his dimensions are recreated using only ten variables. The two dimensions are (1) traditionalism vs. secular-rational authority and (2) survival vs. well-being [12, 30, 31]. In 2005, Inglehart and Welzel teamed up to create their influential cultural maps for which parts of the methodology and indexes were released to the public in 2019 [31, 32]. In 2010, Welzel performed his own analysis which continued the work of Inglehart and hypothesized his own five dimensions related to self-expression based on the WVS data [33]. To date, studies attempting to replicate the Inglehart-Welzel dimensions have been unsuccessful, resulting in some researchers questioning whether questions are truly measuring the same values across countries [8, 34, 35].

Beyond how questionnaires were developed, many of the existing cultural models are criticized for the representativeness of their samples. For example, Hofstede’s model has questionable generalizability since the survey data is from primarily male, highly-educated employees of IBM in the 1970s [36]. Similarly, Schwartz based his analysis on the study of teachers and students [14] and the GLOBE study surveyed organizational leaders [7]. In all three of these models, the dimensions have been generalized to entire countries. Inglehart and colleagues who use the World Values Survey are creating models that are based upon nationally representative samples [29].

In all of these studies, individuals complete the questionnaires and results are averaged either before analysis or the dimension scores are averaged after analysis to create a country mean. Country means are country-level data points, but they cannot be used to make specific conclusions about individuals. This type of data is often classified as an ecological measurement because the level of analysis changes from individuals to countries [2, 37, 38]. Unfortunately, polytomous and binary questions cannot be averaged into a meaningful number; therefore, these questions are reduced to a percentage prior to analysis. For example, when Hofstede measures Power Distance he included the percentage of respondents who preferred consultative managers [2]. With the exception of the Global Leadership and Organizational Behavior Effectiveness (GLOBE) model, all currently available country cultural models aggregate questions to the country level before calculating dimensions [2, 8, 11, 39]. These types of aggregation limit available models because they not only lose details about response patterns and the variability of the responses within a country, but also may dilute the between-country variation with the within-country variation [40]. The GLOBE model uses a multilevel factor analysis, grouped by country to analyze and understand the results of their questionnaire before aggregating to a mean dimension score for each country [7]. The GLOBE country scores for each dimension are averages of the relevant dimension questions, likely due to limitations in the methodologies and computing abilities at the time [7, 8, 39, 40]. Called convergent-emergent constructs by multi-level researchers [41], grouping individual responses by organizational or in this case country groups allows collective opinions and values to emerge for each country from the data rather than assuming the average represents a collective opinion [42]. Following a similar methodology, this study also follows a multi-level approach to discover national social dimensions.

## Current study

The current study produces a new set of social dimensions for 56 countries. Similar to Georgas et al [43], the social dimensions studied in this paper are defined as psychological variables that vary at the national level. For multi-level researchers, our social dimensions are convergent-emergent constructs [41, 42]. The discovered social dimensions are convergent because they are based on survey data from individuals in different countries that we believe can be represented by a single mean. These social dimensions are also emergent because the properties of these dimensions are discovered at the national level. Finally, we refrained from labeling these dimensions as cultural because they have not been studied over time; however, these dimensions should not be used to predict individual values. Instead, these dimensions should be used to understand trends in national context variables where psychological values may play a role, such as the percentage of females attending schools or the percentage of renewable electricity in a national grid.

The study began with the primary goal of creating a model of social differences that was representative and reproducible unlike the current models available. Due to advancements in statistical programming and computing power [44], our second goal was to account for the distribution of responses within a country using a multilevel and multidimensional item response model. We do not believe that nations are culturally or socially homogeneous; however, we do agree that values will tend to differ between countries and provide valuable insight [45]. Using countries to define groups for this study does not deny the existence of subcultures. It instead presents the characteristics of culture that are apparent across countries and assumes that countries vary more between countries rather than within the country [46]. Hofstede and collaborators demonstrated that even though national cultural parameters mask unique traits of regional (sub) cultures, these subcultures tend to cluster as a country [45]. Thus, social dimensions can indicate general trends and shared interpretation within a country and should be used to compare countries rather than examine the specific score of a particular country. Finally, our last goal was to discover the questions that change most meaningfully across countries and eliminate questions which do not show meaningful differences. We have done so by using a data science approach and a multilevel analysis. Based on the existing quantitative cultural models, we expect that there to be between four and seven social dimensions emerging from the data. Moreover, we do not expect this study to uncover values that had never been measured or proposed before; instead we aim to create a model that more accurately represents those values and builds on previous models by offering improved transparency and reproducibility using a big data approach.

## Method

**Fig 1:**
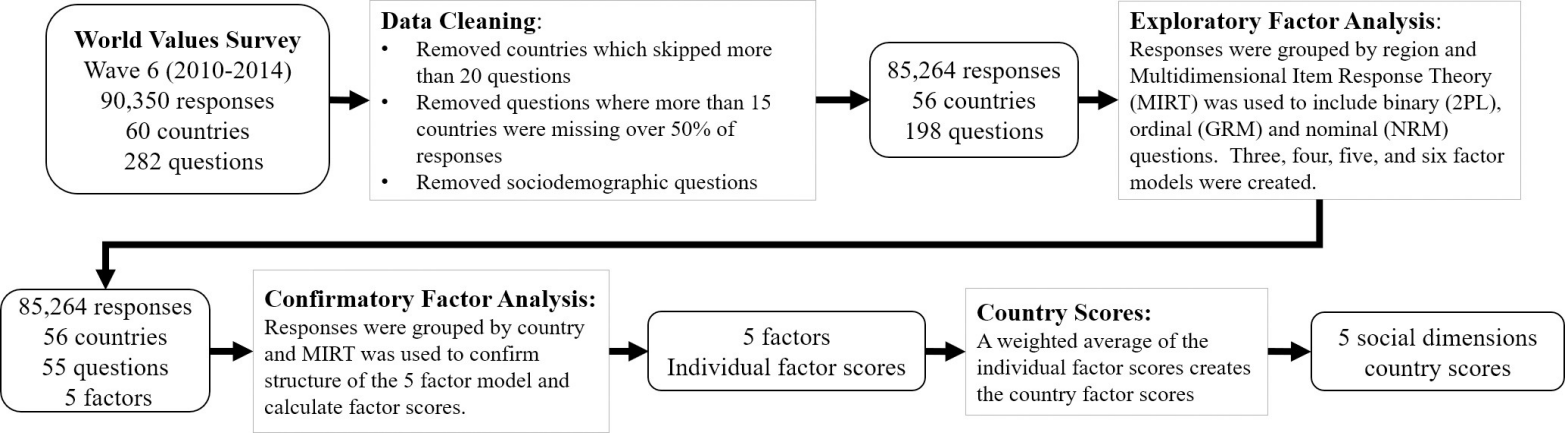
Methodology.

Similar to big data studies, this study uses a data science approach to complete an exploratory statistical analysis and discovers national social dimensions embedded in the responses collected by the WVS. Big data is a relatively new resource for researchers, and it requires a new set of analysis skills that the academic world is just beginning to understand [47, 48]. This study contains information collected from 90,000 people from around the world. Our analysis identified statistical relationships within 198 questions across 56 countries. Fig 1 provides a summary of the methodology. To provide more details, we start by describing the WVS data. Then in the analysis subsection, we describe multidimensional item response functions, which are applied in an exploratory and confirmatory factor analysis. Finally, we describe how factor scores of each social dimension were created for each country. The social dimensions discovered are explained in the results section.

### Data

This study uses data from Wave 6 of the World Values Survey. As downloaded, there are 339 coded items from 60 countries (accessible at www.worldvaluessurvey.org). The questions are a combination of ordinal (interval), nominal (unordered), and metrical responses. The data had a significant number of missing values that had to be dealt with before data analysis. We chose not to remove all responses with any missing values because over half of the data would be eliminated from the data set. Instead, four countries are removed from the analysis due to large amounts (over 50%) of missing values for more than 20 questions, and 42 questions are removed from the analysis due to large amounts (over 50%) of missing values for 15 countries. All questions related to sociodemographics were also removed from the dataset because these questions do not represent values. All questions that had open-ended responses such as political affiliation or religious denomination are also removed. Finally, we remove questions asking about the respondent’s environment; the responses to these questions are not choices that the respondents can make based on their values. For example, the question “how frequently do robberies occur in your neighborhood” was removed because responses depend on the respondents’ living environment not on values. The resulting dataset contains 56 countries (85,264 individual respondents) and 198 question variables. The final step of data cleaning involved transforming responses to particular questions into smaller numbers of response categories in order to reduce the computational requirements. For example, questions answered on a scale from one to ten were reduced to a scale of one to five. The R scripts used in this analysis can be found at https://github.com/Laalliso/SocialDimensions. To download the WVS wave 6 data, please visit http://www.worldvaluessurvey.org/WVSDocumentationWV6.jsp

### Analysis

To reduce the data set into a series of latent factors, several multidimensional item response models were created. Unlike manifest variables (e.g. age), latent factors (e.g. happiness or the social dimensions of interest in this study) cannot be directly measured. Therefore, latent factors are typically measured through a combination of manifest variables and are fundamentally based on the manifest variables that are included in the analysis. These latent factors are new continuous variables [49]. Multidimensional item response is similar to a factor analysis, but it differs in a few key aspects. Factor analysis is a model-based technique that aims to explain the correlations between continuous variables. In contrast, an item response model uses a function to model the probability of a single response being in a response category, often called the item response function [49]. For example, the item response function for binary data uses a logit function similar to what is used in logistic regression to link binary data to a continuous probability. Thus for item response functions, the input variables may be binary, ordered, categorical, and/or nominal. The questions in the WVS are binary, ordered, or nominal; therefore, we use item response functions to model the probability of answering in a response category for a question [49, 50]. In both cases, the output is a continuous latent factor. In the following section, we explain more details of item response functions and then we explain for item response functions are used in exploratory and confirmatory factor analysis.

#### Item response functions

The probability of a certain response for the item, *i*, given the latent factor is denoted as *π_i_*(*f*) as seen in Eq 1 below. In this analysis, the items are the questions from the WVS. Note that Eq 1 was developed for the dichotomous case; additional functions must be added when there are more than two categories, as explained below [49].

$$\pi_{i}(f)=\frac{1}{1+\exp\left(-\left(\alpha_{i0}+\sum_{j=1}^{q}\alpha_{ij}f_{i}\right)\right)}$$ (1)

The item parameter that measures easiness (*α*_*i*0_) shifts the item response function left and right. The discrimination parameters (*α*_*i*0_…*α_iq_*) alter the steepness of the curve, altering the relationship between the factor values and the likelihood of a response for the observed variable. This model is called the two-parameter logistic model (2PL) due to the two parameters describing the response function. We use this model for questions with only two responses (i.e. dichotomous variables) [49, 51].

When there are multiple categories, we use a cumulative approach for the ordered responses, and the nominal approach for the unordered responses. The cumulative approach estimates the probabilities of a particular response based on the difference between two adjacent cumulative response probabilities. In item response theory, the graded response model (GRM) is a widely used cumulative method. The graded response model replaces the category response function *π_i_*(*f*) with the cumulative response function *γ_i(s)_*(*f*). The probability of a response being in a particular category is calculated as the difference between the probability of a response being in a category greater than or equal to the category of interest and the probability of a response being in the category greater than or equal to the response category above of the category interest [49, 52, 53]. Eq 2 applies given a certain latent ability (θ) and a set of parameters (ψ) applied to both k and k+1. Probability is estimated using the equation for *π_i_*(*f*) shown above [52].

P(x=k∣θ,ψ)=P(x≥k∣θ,ψ)−P(x≥k+1∣θ,ψ) (2)

The nominal response model (NRM) is used for nominal responses. This model is a generalized version of the general partial credit model [54, 55]. Instead of comparing the cumulative probability of one response to the one adjacent to it, this model compares the probability of a single category against a reference category using a multinomial logistic function [51, 56]. The NRM has two parameters explaining how the latent factor(s) relates to the probability of a certain response as seen in Eq 3 below. However, since there are now unordered categories the discrimination parameter is divided into two different coefficients, one for the entire item or question and another representing the specific response. These are called the scoring coefficients (*ak_i_*), and they represent the probabilistic ordering of the categories and aid with interpretation of the model (Chalmers, 2018).

$$P(x=k|\theta ,\psi )=\frac{\exp\left(ak_{k-1}*\left(a_{1}*\theta_{1}+a_{2}*\theta_{2}+\cdots +a_{q}*\theta_{q}\right)+d_{k-1}\right)}{\sum_{1}^{k}\exp\left(ak_{k-1}*\left(a_{1}*\theta_{1}+a_{2}*\theta_{2}+\cdots +a_{q}*\theta_{q}\right)+d_{k-1}\right)}$$ (3)

The statistical program R was used to estimate these item response functions for our data. Specifically, we used version 1.29 of the *mirt* package [44]. The *mirt* function uses marginal maximum likelihood estimation with the expectation-maximization (EM) algorithm to estimate the item response function parameters as well as the correlations between the proposed latent factors [44, 49]. With the *mirt* function we specified 2PL for questions that had only two response categories or where only two response categories were used, GRM for questions with ordered responses, and the NRM for questions with unordered responses. The expectation-maximization algorithm is an iterative process where an initial set of parameters are randomly chosen, and expected distributions for different response patterns are created based on the values of the latent factors [57]. New parameters are selected to maximize the marginal likelihood that the parameters create the observed response patterns. The process iterates until the model parameters converge for all item parameters [49, 58–60]. To avoid indeterminacy, the distribution of the latent factor in this analysis is normally distributed [49, 61]. Finally, the estimation process requires the number of a factors to be estimated. We determined the number of a factors through several exploratory factor analyses.

#### Exploration: Exploratory factor analysis

Any exploratory technique looks for relationships within data without prior assumptions about relationships. Similarly, an exploratory factor analysis (EFA) does not require a model structure containing the pattern of potential associations between the manifest variables and the latent factors. In this way, an EFA searches for the best model structure between the manifest variables and one or multiple latent factors. Since item response functions are being used to model the latent factors, the analysis determines a set of item parameters that is most likely to predict the outcome of a particular question based on a latent factor. For example, if the latent factor is fairness and the question asks a respondent if stealing is justifiable on a scale of one (always justifiable) to five (never justifiable), the item response function is looking for item parameters that are able to best estimate the probability of being in a certain response category, such as “never justifiable”. The parameters for each item are converted into traditional factor loadings. This approach has also been called an item factor analysis [62]. EFAs do require the number of latent factors to be estimated. For our analysis, three, four, five, and six factor models were created based on the 198 questions from the WVS. The questions included in the exploratory analysis can be found in the S1 Table.

Each exploratory factor analysis is constrained (1) to grouping the responses by country and (2) for the item parameter values to be equal across the different countries. The factor means are freely estimated for different countries, while the covariance is held at one. This grouped analysis into consideration the distribution of each country when selecting the best parameters for item response functions. In technical terms, this model assumes that the WVS survey questions are invariant across countries, and the effect of group membership, also called an impact, can be shown by differences in the latent factors. Impact studies are focused on the true group differences rather than inconsistencies within the questions [63]. Unlike measurement invariance or bias, the goal of impact studies is to understand the differences between groups. Thus, the model is constrained to look for differences that are present across countries. The item parameters are translated into factor loadings, and factor loadings are interpreted as correlations between the latent factor and the manifest variables or questions in this analysis [58, 62]. The factor analysis becomes multidimensional when more than one latent factor is modeled. In our analysis, we tested item response models ranging from one to six factors. In an EFA, the factor loadings can then be rotated to clarify interpretation. Rotations are often used to clarify interpretations [49]. Rotating the latent factors does not change the model but allows new perspective on the same latent factors. For example, the oblimin rotation allows for the model to have correlated factors, but does not require it [64]. Based on the Akaike information criterion (AIC) and the Bayesian information criterion (BIC), the five-factor model is selected as the best fit model. AIC assumes that a true model does not exist and instead focuses on how well the model predicts future data while BIC looks for models with the highest probability of being the true model. The best model minimizes both values [65, 66].

#### Validation: Confirmatory factor analysis

Unlike an EFA, a confirmatory factor analysis (CFA) has a predefined model structure, meaning that the relationships between the manifest variables and latent factors are identified before the analysis starts. The model can no longer be rotated because the relationship is defined in the model structure. A CFA confirms the structure of the latent factors seen in the EFA and allows for the calculation of a series of absolute fit parameters that demonstrate if the CFA model is a good fit of the original data [49, 67]. To measure how well the data fits into the model, several absolute fit parameters are used. Root mean square error of approximation (RMSEA) estimates the ability of the model to exactly estimate the sample rather than approximately. Values under 0.05 are considered a good fit; while 0.1 is considered a poor fit. The Comparative Fit Index (CFI) and the Tucker Lewis Index compare the fitted CFA model with a null model where the variables are not correlated. CFI and TLI values closer to 1 indicate improved fit [49, 68].

Using the relationships that had previously emerged in the EFA, we define a CFA model containing 55 questions of the original 198 questions. All 55 questions have loadings of 0.3 or higher onto at least one of the five latent factors from the oblimin rotation of the EFA. We tested CFA models with all of the factors correlated and uncorrelated. Unfortunately, we are unable to complete a CFA with all of the factors correlated. Since we subdivide our data into 56 groups (one for each country), each sample becomes relatively small for the number of item parameters that we are estimating. Therefore, the latent factor variance matrix becomes non-positive, and the estimation fails. The estimation is successful when the latent factors are assumed to be uncorrelated, similar to previous studies which maintain that latent factors should remain uncorrelated [2, 12, 37]. (See the S2 Table in the supporting information for the 55 questions and final factor loadings).

### Factor scores

The final step is to estimate the factor scores. Factor scores are estimated using maximum a posteriori (MAP) which selects the factor score based on the maximum posterior density for the given response pattern [69]. To estimate the factors scores for each respondent, the item response functions were evaluated between negative six and six. Since we are interested in a country score, we calculated a weighted average of all the factor scores within a country to create a country score for each latent factor. In the WVS, each individual respondent is given a weight so that each sample can be adjusted to accurately represent a country population. Each country was allowed to determine how the sample should be weighted to represent their country; the majority of countries relied on gender, age, education, and socioeconomics to adjust samples [70]. The factor scores were multiplied by 10 so that individual factor scores range from -60 to 60.

## Results

Six EFA models are created using the *mirt* package (v1.29) in R (Chalmers, 2018). Each model has a different number of exploratory factors, ranging from one to six factors (also call latent dimensions). Each model has 56 groups, one for each country. Item parameters are constrained to be equal across groups. The five-factor exploratory model has the lowest AIC and BIC of all the models created and so is discussed. We utilize high-performance computing in order to run these models. Exploratory models above four factors were exceptionally large (over 250GB); the size of the model is reasonable considering that it contains 56 groups and 198 items with a series of responses that must be modeled independently as item response curves (Chalmers, 2012). The outputs of these models are series of item parameters that have been converted into factor loadings [62]. Loadings represent how the question (often called an item in IRT literature) relates to the factors and range from -1 to 1. Analyzing the factor loadings allows us to name the factors. These five factors are quantitative representation of social dimensions. From here forward, we refer to the discovered five factors as social dimensions and provide an interpretation of each social dimension in the discussion.

Table 2 below shows the top five questions that load on each social dimension. The first social dimensions (SD1) is based on 11 questions. The second social dimension (SD2) is based on 14 questions. The third social dimension (SD3) is based on 7 questions. The fourth social dimension (SD4) is based on 18 questions. Finally, the fifth social dimension (SD5) is based on 6 questions. For the full list of the questions used to measure each social dimension, please see the S2 Table included as supporting information. We repeated the CFA several times to determine the stability of the model under different estimation methods. As expected the results numerically vary slightly [71]; however, the countries’ scores relative to each other (i.e. rank) remain consistent across the different estimation methods. The numeric results in this paper are from the CFA model with the best global fit values, shown in Table 3. The loadings are estimated using the traditional EM method described in the methods section. Our CFA models converge when the max change in the item parameters is less than 0.0005.

**Table 2:** Confirmatory Factor Analysis (CFA): Top 5 question loadings on social dimensions.

Factor	QUESTION	Loading
SD1	Religion is very important in your life	-0.61
	Believe in God	-0.61
	Strongly agree that whenever science and religion conflict, religion is always right	-0.51
	You pray several times a day	-0.47
	Religious faith was mentioned as a quality that children can be encouraged to learn at home.	-0.44
SD2	Not members of an environmental organization	-0.76
	Not members of a consumer organization	-0.76
	Not members of a humanitarian or charitable organization	-0.74
	Not members of a professional organization	-0.72
	Not members of a self-help or mutual aid group	-0.76
SD3	Stealing property is never justifiable	-0.77
	Someone accepting a bribe in the course of their duties is never justifiable	-0.74
	Cheating on taxes if you have a chance is never justifiable	-0.70
	Violence against others is never justifiable	-0.63
	A man to beat his wife is never justifiable	-0.58
SD4	You have no confidence at all in Parliament	-0.77
	You have no confidence at all in the government in your nation's capital	-0.74
	You have no confidence at all in the civil service	-0.70
	You have no confidence at all in political parties	-0.63
	You have no confidence at all in the courts	-0.58
SD5	Not at all worried about a civil war	0.82
	Not at all worried about a terrorist attack	0.80
	Not at all worried about a war involving your country	0.80
	Not at all worried about not being able to give your children a good education	0.49
	Not at all worried about government wire-tapping or reading my mail or email	0.47

**Table 3:** Absolute fit values at global level for CFA model.

	N	DF	RMSEA	TLI	CFI
CFA_Global_Data	85264	1337	0.0661	0.884	0.889

Table 3 shows the global fit for the CFA model. There is not enough data available to calculate the fit of the model within each country; therefore, these fit values were calculated with all the data (global level). As described in the methods section, RMSEA values should be less than 0.05 while CFI and TLI should 0.95 for good fit. We believe our slightly higher values for RMSEA and slightly lower CFI and TLI values could be due to the global data analysis.

The final output of the model are a set of factor scores. Factor scores estimate a respondent’s position (or rank) within the social dimension. The factor scores are calculated using the loadings from CFA [72]. The weighted average of the factor scores within a country is the final country score. The S1 File in the supporting information shows the number of responses per country for each of the 55 questions in the CFA. Each country sample is a similar size. This ensures that no single country dominates the data just because it contains more responses.

**Fig 2:**
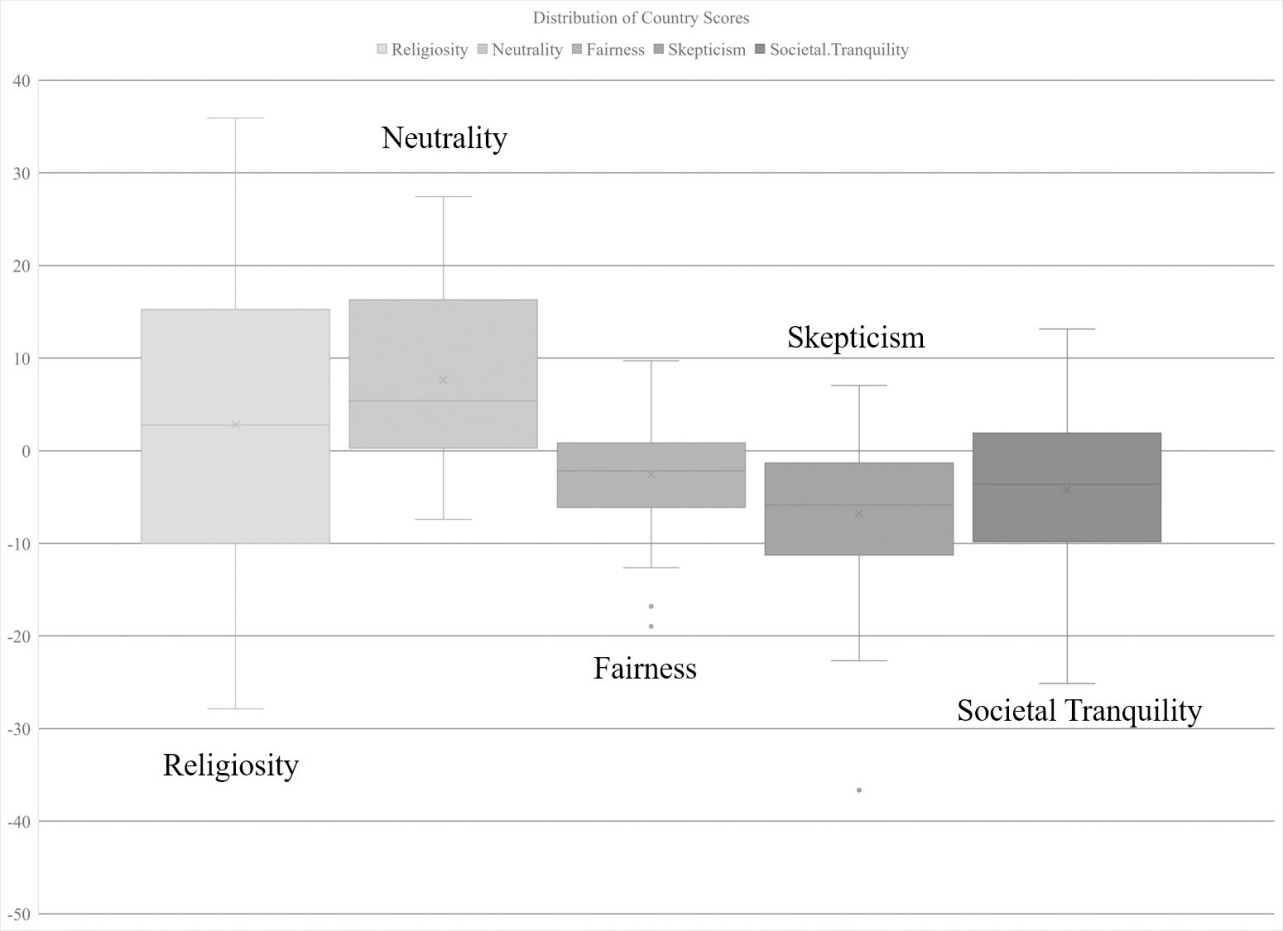
Distribution of country scores on social dimensions.

The country scores are useful in understanding how our new social dimensions relate to existing cultural dimensions as well as other national context variables as presented in the next section. The spread of country scores is shown in Fig 2. A list of country scores for all 56 countries on each social dimension can be found in Table 4 or in the S2 File of the supporting information.

**Table 4:** Country scores for each social dimension.

WVS	Country Name	Religiosity	Neutrality	Fairness	Skepticism	Societal Tranquility
Country Code						
12	Algeria	26.40	16.29	-12.62	-1.68	-10.60
32	Argentina	-8.34	4.61	-2.80	2.29	6.45
51	Armenia	7.52	23.23	2.01	0.47	-14.95
36	Australia	-20.17	-2.39	0.19	-5.78	5.58
31	Azerbaijan	0.75	27.45	5.36	-10.22	-6.29
112	Belarus	-5.59	5.61	-5.33	-7.68	-1.97
76	Brazil	3.00	2.48	-1.59	-1.53	-4.89
152	Chile	-6.98	4.02	-1.63	-3.33	2.59
156	China	-22.84	19.78	-6.46	-22.69	-0.72
170	Colombia	7.33	0.55	-1.43	-1.95	-14.82
196	Cyprus	-0.22	5.77	2.04	-7.68	-0.55
218	Ecuador	5.95	10.79	-2.63	-3.03	-9.79
233	Estonia	-19.08	11.98	-2.55	-11.64	-0.39
268	Georgia	16.06	20.86	8.99	-2.07	-17.12
276	Germany	-16.98	2.77	0.51	-8.65	6.78
288	Ghana	22.05	-0.81	0.57	-18.23	-14.37
344	Hong Kong SAR, China	-13.08	1.04	-7.84	-13.18	4.01
356	India	11.21	-1.58	-5.80	-18.10	-3.19
368	Iraq	23.05	16.64	-6.92	-0.62	-5.77
392	Japan	-17.40	8.52	6.97	-7.26	-9.81
400	Jordan	35.93	17.13	2.30	-5.13	0.73
398	Kazakhstan	-6.17	16.38	-6.52	-12.05	-6.39
417	Kyrgyz Republic	9.87	5.03	-6.03	-10.68	-9.87
422	Lebanon	6.05	2.51	-11.02	0.04	-5.76
434	Libya	33.91	9.93	-1.43	-0.47	-13.13
458	Malaysia	17.24	9.43	-6.42	-18.78	-18.98
484	Mexico	2.73	-0.57	-7.09	-1.00	-17.29
504	Morocco	26.57	19.72	0.77	-10.05	-1.84
528	Netherlands	-25.33	1.20	3.01	-4.40	12.52
554	New Zealand	-16.03	-4.50	1.04	-8.56	8.90
566	Nigeria	23.62	-4.35	-5.23	-9.51	-8.30
586	Pakistan	30.30	13.16	0.36	-3.07	-5.64
275	Palestine	27.02	8.98	-6.89	-0.15	-1.89
604	Peru	2.79	4.36	-5.32	5.35	-9.46
608	Philippines	12.30	-1.90	-16.80	-17.54	-11.24
616	Poland	0.46	9.71	-1.49	-1.37	1.60
642	Romania	6.81	13.56	5.39	2.32	-2.20
643	Russian Federation	-10.63	19.02	-7.59	-1.61	-4.07
646	Rwanda	3.85	-6.06	-5.77	-12.32	-20.60
702	Singapore	-1.28	5.17	-8.70	-19.02	2.96
705	Slovenia	-17.84	4.96	-0.13	4.09	5.51
710	South Africa	5.81	-7.40	-18.97	-7.62	-0.19
410	South Korea	-9.79	2.68	-2.79	-8.39	-3.02
724	Spain	-17.93	10.59	1.32	-1.14	-0.58
752	Sweden	-27.89	-2.80	-4.11	-11.15	13.13
158	Taiwan	-6.54	-4.37	-3.26	-9.08	-3.98
764	Thailand	-0.55	0.95	-0.79	-12.72	-1.13
780	Trinidad and Tobago	12.35	-0.51	4.12	-3.53	2.77
788	Tunisia	26.04	23.66	-2.23	7.05	-25.13
792	Turkey	16.78	20.69	9.71	-10.09	-5.05
804	Ukraine	-5.00	16.86	-5.70	-0.04	-4.34
840	United States	-3.27	-2.99	-2.10	-3.71	2.39
858	Uruguay	-13.92	9.87	2.30	-5.95	1.76
860	Uzbekistan	0.94	17.83	-3.04	-36.66	3.86
887	Yemen, Rep.	25.46	14.53	-1.91	7.00	-13.87
716	Zimbabwe	15.00	-1.72	-6.90	-12.10	-6.77

## Discussion

In this discussion, we first name each of the five social dimensions and second present a comparison with previously published cultural models. We estimate each country’s position along the social dimensions using factor scores. It is important to note that lower scores do not indicate poor results; instead, they represent a different social view than countries with higher scores. Similarly, countries with high scores are in no way superior to those with lower scores; the countries simply are at the opposite end of the scale for that social dimension.

### Five discovered dimensions

The first social dimension (SD1) contains questions related to belief in God and support of religious customs as seen in the first five rows of Table 2. We have labeled this first social dimension as **Religiosity** because it represents a strong dedication to God and religious customs, but not any particular religious denomination. This dimension focuses on foundational moral values that historically have been held by religious leaders and texts [73]. Furthermore, this dimension supports the findings of Saucier et al. (2011) who aimed to identify questions that most significantly changed between countries [74]. Examples of the WVS questions include disapproval of divorce and homosexuality, opposition to abortion, the belief that religion is always right, the belief that religious leaders should be a part of the government, as well as confidence, membership and attendance in religious groups. The Religiosity dimension means that people in countries with high scores such as Jordan, Pakistan, and Libya tend to be more traditional in following religious customs and beliefs while people in countries with low scores such as China, Netherlands, and Sweden tend to be more accepting of secular and alternative lifestyle choices.

The second social dimension (SD2) measures **Neutrality** represented by questions related to membership (or lack thereof) in various types of organizations as well as participation in political activities. Neutrality represents low engagement with both civic engagement groups as well as individual needs or interest groups. Questions that load on to the factor include non-membership in environmental organizations, self-help groups, political parties and sports organizations. Neutrality also includes a lack of motivation to become involved in civic or political issues with the questions about participation in boycotts, sticks, and petitions. The countries with the highest scores (i.e. Azerbaijan, Armenia, and Tunisia) are countries where over 95% of the population are not in social organizations such as environmental organizations, humanitarian or charitable organizations. Neutrality therefore represents a lack of engagement and relevance of such activities [75]. Countries with low Neutrality scores (i.e. South Africa and New Zealand) have more participation and therefore voluntary organizations have more relevance in these countries. Still, it is important to note that even low-scoring countries have just half to one-third of its citizens in these social groups.

The third social dimension (SD3) represents **Fairness**. As seen in Table 2, the questions that load on to this factor ask if actions such as stealing, bribery, and violence are ever justifiable, despite the fact that these are illegal in the majority of justice systems. Theoretically, fairness is associated with a judgment of a social situation that creates cooperative behaviors; it can be used both proactively to create cooperative behaviors or reflectively to judge previous actions [76, 77]. This dimension does not define what constitutes stealing, bribery, or violent actions; however, it does identify the importance a society places on avoiding such behaviors as they are locally defined. The countries with high scores for Fairness such as Turkey, Georgia, and Japan have more than 85% of the population stating that it is never justifiable to do these things. Countries like South Africa, Philippines, and Algeria, have lower scores for Fairness, meaning approximately 50% of the population believe that it is never justifiable to do things like stealing, bribery, or violence.

The fourth social dimension (SD4) represents **Skepticism**. This dimension measures the lack of confidence in large organizations and institutions [78]. Skepticism specifically identifies an unwillingness to give organizations (political or civic) the benefit of the doubt [79]. Skepticism is considered a vital part of a democracy because it ensures that people continue to stay involved with elections and decisions made by the government. Skepticism holds organizations accountable by other organizations as well as court systems [80]. Peru, Yemen, and Tunisia have high Skepticism scores, implying that large proportions of the population (30% or more) tend to have relatively less confidence in their major institutions such as government groups, labor unions, press, and major corporations. On the other hand, Uzbekistan, China, and Singapore have much lower Skepticism scores, with over 70% of the population having relatively more confidence in institutions.

The fifth and final social dimension (SD5) represents **Societal Tranquility**. This dimension measures the lack of worry related to societal events and conversely the importance of peacefulness. Worrying is a symptom of anxiety due to uncertainty [81, 82] and how comfortable societies are with these uncertainties. Some worries are associated with circumstances such as civil war that may be out of respondent’s control [81] and are therefore experienced at the societal level. Therefore, worrying can be imposed at the societal level [83]. Societal Tranquility is peace of mind that people have learned to value such that people feel safe, calm, and harmony in the same way across countries [84, 85]. Policies may address this dimension; for example, in the preamble to the U.S. constitution, domestic tranquility is established as a fundamental value of the United States and represents the importance of rejecting injustice and violence, such that individuals are protected from harm [84, 85]. In countries such as Sweden, Netherlands, and New Zealand approximately half of the responses indicated that they worry very little about war, terrorism, spying, employment, or education for their children (top five questions per Table 2), demonstrating Societal Tranquility. On the other hand, in Tunisia, Rwanda, and Malaysia, 80% of the respondents are worried about those top five issues, with the exception of government spying where only 60% of the people are worried. It is worth noting again that historical and current situations (i.e. civil war, economic growth) within a country undoubtedly provide substantial context for the concerns in this dimension; nevertheless, it is still a social value if societies worry about these situations.

### Comparison with existing models

Table 5 shows how our discovered social dimensions relate to the currently available and published cultural dimensions mentioned in Table 1. Each of the five social dimensions described here correlates with at least one dimension described by Hofstede [86], Inglehart & Welzel [29], Schwartz [18], or GLOBE [87]. However, none of the discovered social dimensions in our model validate a previous social or cultural dimension as seen by multiple correlations between each social dimension and multiple existing dimensions. For example, Societal Tranquility is significantly correlated with three of Hofstede’s six dimensions, both of Inglehart’s dimensions, three of Schwartz’s seven dimensions and seven of GLOBE’s 18 dimensions. Nevertheless, these correlations provide significant insight into validating our new dimensions with theory. For instance, Religiosity is directly correlated to not only embeddedness (defined as the maintenance of social order [88]), but also power distance (defined as the imbalance of power [2]) and in-group collectivism (defined as the pride in group structure [7]). Similar to a religion embeddedness, power distance, and in-group collectivism support social hierarchy, providing community and discouraging selfish actions.

**Table 5:** Country score correlations with published cultural dimensions (Pearson).

		# of Countries	Religiosity	Neutrality	Fairness	Skepticism	Societal Tranquility
Hofstede	Power Distance	34	0.49**	0.41*	-0.43*		-0.68***
	Individualism	34	-0.48**				0.56***
	Masculinity	34					-0.45**
	Uncertainty Avoidance	34			0.47**	0.76***	
	Long Term	47	-0.52***				
	Indulgence	47		-0.59***			
Inglehart/Welzel	Self-Expression	54	-0.72***	-0.48***			0.56***
	Secular	54	-0.68***				0.40**
Schwartz	Harmony	38	-0.43**				
	Embedded	38	0.83***				-0.68***
	Hierarchy	38				-0.50**	
	Mastery	38				-0.35*	
	Affective Autonomy	38	-0.63***				0.66***
	Intellectual Autonomy	38	-0.71***		0.35*		0.46**
	Egalitarianism	38				0.34*	
GLOBE—Practices	Uncertainty Avoidance	30		-0.41*		-0.56***	0.37*
	Future Orientation	30		-0.39*		-0.51**	0.09
	Power Distance	30	0.65***				-0.49**
	Instit. Collectivism.	30	-0.41*			-0.49**	
	Humane Orientation	30				-0.50**	
	Perform. Orientation	30		-0.41*		-0.50**	
	In.group Collectivism	30	0.65***	0.41*			-0.65***
	Gender Egalitarianism	30					
	Assertiveness	30					
GLOBE-Values	Uncertainty Avoidance	30	0.57**				-0.63***
	Future Orientation	30	0.73***				-0.48**
	Power Distance	30				-0.46*	
	Instit. Collectivism.	30	0.41*				
	Humane Orientation	30					
	Perform. Orientation	30		-0.46*	-0.39*		
	In.group Collectivism	30					
	Gender Egalitarianism	30	-0.38*	-0.46*		0.46*	0.38*
	Assertiveness	30				-0.46*	

(Significance codes: ‘***’ 0.001 ‘**’ 0.01 ‘*’ 0.05).

Interestingly, Hofstede’s uncertainty avoidance index strongly correlates with Skepticism rather than Societal Tranquility, suggesting as others have that uncertainty avoidance lacks reliability [27]. Similarly, Hofstede’s long term orientation and indulgence dimensions are based on six questions from the World Values Survey [9]; however, none of those questions were identified by our analysis, which indicates that they do not show meaningful differences between countries and supports new claims by Minkov that long term orientation should be updated [28]. Therefore this study fails to validate existing models, suggesting that current measurements of social differences may in fact be a combination of social and cultural values [38] and that the discovered social dimensions provide updated, empirical measurements for social dimensions.

### Demonstration of the social dimensions’ utility

The correlations between the social dimensions and frequently used national-level variables as listed in Table 6 (GDP per capita, Suicide Rate, Global Innovation Index, Human Development Index, Corruption Perception Index, and the Polity Index) demonstrate the future utility of these social dimensions for other researchers. For example, the strongest negative correlation is between the Human Development Index (HDI) and Religiosity, suggesting that, similar to previous research [89], countries with stronger religious values tend to score lower on the HDI. This relationship has been studied before by Inglehart and colleagues; however, this study suggests that it is religion which correlates with a nation’s HDI score rather than broad “traditional” values that Inglehart identified from the WVS [12]. The strongest positive correlation was found between Societal Tranquility and GDP per capita, implying that countries with more wealth per person generally place more importance (and likely resources) on the establishment of peace. Again, this relationship provides a specific insight into Inglehart’s perspective on modernization theory. Whereas Inglehart and collaborators suggested that growth in the wealth of correlates with a change in both of his dimensions, evidence shown in Table 6 suggests that it is primarily Religiosity and Societal Tranquility. Finally, the Polity Index places a country along an autocratic-to-democratic continuum [90] and is related to all five of the discovered dimensions. This relationship confirms previous research that democracy is impacted by values [91, 92] and again specifies that some values (e.g. Neutrality) may play a more important role than other values. However, as with all zero-order correlations it is important that future research should investigate how control variables impact these relationships.

**Table 6:** Country score correlations with national context variables (Spearman correlations).

	# of Countries	Religiosity	Neutrality	Fairness	Skepticism	Societal Tranquility
GDP per Capita^1^	55	-0.73***				0.62***
Suicide Rate (2015)^2^	54	-0.67***	-0.24 ^			0.45***
Global Innovation Index^3^	52	-0.73***				0.60***
Human Development Index^4^	55	-0.77***		0.24 ^		0.61***
Corruption Perceptions Index^5^	55	-0.53***	-0.34*	0.34*		0.51***
Polity Index^6^	54	-0.44***	-0.48***	0.30*	0.24 ^	0.41*

(Significance codes: ‘***’ 0.001 ‘**’ 0.01 ‘*’ 0.05 ‘^’ 0.1).

*All national indicators are from 2014 except for the suicide rate which was only measured in 2015*.

1- [93], 2- [94], 3- [95], 4- [96], 5- [97], 6- [90].

### Limitations

Our data science approach required several choices and assumptions which impact our results. First, we use a pre-existing dataset for which we did not specify the questions or sample size. We therefore are limited to the questions included in the existing WVS survey, which covers not only ethical and political views, but also behaviors that could be considered context specific (e.g. joining in boycotts, worrying about civil war). Previous research of personally (rather than nationally) measured value-behavior relations suggests that while personal values motivate behavior the relationship and causal mechanisms can be influenced by normative pressures [98]. We assume that these normative pressures are created by the latent value dimensions that we have measured in this analysis. As such, some dimensions may be influenced by historical context. Indeed from the perspective of the ecocultural framework, Societal Tranquility suggests that eco-social indices (such as the time since a civil war or terrorist attack) may be insightful variables in future studies. Furthermore, future studies should continue to investigate and experiment with additional questions related to the theoretical foundation of the five social dimensions identified in order to gain a deeper understanding of the identified social dimensions.

Second, we chose to use a multi-level analysis that assumed countries can be defined by a single value for each social dimension. The country scores presented in this study allow us to understand where a country ranks on a specific social dimension; however, it should never be assumed that all individuals within a country follow those trends. Future research should consider how the distribution of individual scores from a single country can be used to more accurately show how a country has social variability within its borders.

Finally, this study is limited to 56 countries and a single wave of the World Values Survey. Future studies should consider expanding the analysis to more countries and a broader timeline. This could be done by using the additional waves of the WVS or through new data collection in additional countries. Since this model is based on data from 2010 to 2014, future iterations of this analysis are needed in order to distinguish if situational attitudes would create change over time [38].

## Conclusion

In this study, we leverage high power computing to discover five quantitative social dimensions from 198 questions in Wave 6 of the World Values Survey using multilevel multidimensional item response functions. The five social dimensions identified are (1) Religiosity, (2) Neutrality, (3) Fairness, (4) Skepticism, and (5) Societal Tranquility. In this article, we show how these social dimensions correlate to quantitative cultural dimensions from previously existing research and also to a selection of frequently used national-level variables. This study improves the measurement of social values across countries by taking a data science approach, starting with nationally representative data, providing a reproducible methodology, including within country diversity, and finally, creating an adaptable procedure for future datasets. While all of these improvements have been used in various models, they have never before been applied in a single model of social dimensions. The current study produced a new set of social dimensions measured across 56 countries and was shown to provide meaningful insight into national context variables as well as existing social theory. Today, this is possible thanks to the tremendous effort of the people behind the World Values Survey and thanks to recent advances in computing capabilities.

## Supporting information

S1 Table WVS questions included in EFA. (DOC)

S2 Table WVS questions included in CFA and factor loadings. (DOCX)

S1 File Sample sizes of 55 items in CFA. (XLSX)

S2 File Country scores for each social dimension. (XLSX)
