# Supplementary material for: Religiosity, neutrality, fairness, skepticism, and societal tranquility: A data science analysis of the World Values Survey
Source: PLoS One. 2021 Jan 11;16(1):e0245231. doi: 10.1371/journal.pone.0245231 (PMC7799817; doi:10.1371/journal.pone.0245231)
Supplement: S1 Table — (DOC) [file pone.0245231.s001.doc]

# S1 Table - WVS questions included in exploratory factor analysis (EFA)

| **ID** | **LABEL** | **QUESTION** |
| --- | --- | --- |
| V4 | Important in life: Family | For each of the following, indicate how important it is in your life. Would you say it is: Family |
| V5 | Important in life: Friends | For each of the following, indicate how important it is in your life. Would you say it is: Friends |
| V6 | Important in life: Leisure time | For each of the following, indicate how important it is in your life. Would you say it is: Leisure time |
| V7 | Important in life: Politics | For each of the following, indicate how important it is in your life. Would you say it is: Politics |
| V8 | Important in life: Work | For each of the following, indicate how important it is in your life. Would you say it is: Work |
| V9 | Important in life: Religion | For each of the following, indicate how important it is in your life. Would you say it is: Religion |
| V10 | Feeling of happiness | Taking all things together, would you say you are: |
| V11 | State of health (subjective) | All in all, how would you describe your state of health these days?. Would you say it is: |
| V12 | Important child qualities: independence | Here is a list of qualities that children can be encouraged to learn at home. Which, if any, do you consider to be especially important?: Independence |
| V13 | Important child qualities: Hard work | Here is a list of qualities that children can be encouraged to learn at home. Which, if any, do you consider to be especially important?: Hard work |
| V14 | Important child qualities: Feeling of responsibility | Here is a list of qualities that children can be encouraged to learn at home. Which, if any, do you consider to be especially important?: Feeling of responsibility |
| V15 | Important child qualities: Imagination | Here is a list of qualities that children can be encouraged to learn at home. Which, if any, do you consider to be especially important?: Imagination |
| V16 | Important child qualities: Tolerance and respect for other people | Here is a list of qualities that children can be encouraged to learn at home. Which, if any, do you consider to be especially important?: Tolerance and respect for other people |
| V17 | Important child qualities: Thrift saving money and things | Here is a list of qualities that children can be encouraged to learn at home. Which, if any, do you consider to be especially important?: Thrift, saving money and things |
| V18 | Important child qualities: Determination, perseverance | Here is a list of qualities that children can be encouraged to learn at home. Which, if any, do you consider to be especially important?: Determination and perseverance |
| V19 | Important child qualities: Religious faith | Here is a list of qualities that children can be encouraged to learn at home. Which, if any, do you consider to be especially important?: Religious faith |
| V20 | Important child qualities: Unselfishness | Here is a list of qualities that children can be encouraged to learn at home. Which, if any, do you consider to be especially important?: unselfishness (* In Spanish "generosity") |
| V21 | Important child qualities: Obedience | Here is a list of qualities that children can be encouraged to learn at home. Which, if any, do you consider to be especially important?: Obedience |
| V22 | Important child qualities: Self-expression | Here is a list of qualities that children can be encouraged to learn at home. Which, if any, do you consider to be especially important?: Self-expression |
| **ID** | **LABEL** | **QUESTION** |
| V23 | Satisfaction with your life | All things considered, how satisfied are you with your life as a whole these days? Using this card on which 1 means you are “completely dissatisfied” and 10 means you are “completely satisfied” where would you put your satisfaction with your life as a whole?: |
| V24 | Most people can be trusted | Generally speaking, would you say that most people can be trusted or that you need to be very careful in dealing with people? |
| V25 | Active/Inactive membership: Church or religious organization | Now I am going to read off a list of voluntary organizations. For each organization, could you tell me whether you are an active member, an inactive member or not a member of that type of organization?: Church or religious organization |
| V26 | Active/Inactive membership: Sport or recreational organization | Now I am going to read off a list of voluntary organizations. For each organization, could you tell me whether you are an active member, an inactive member or not a member of that type of organization?: Sport or recreational organization |
| V27 | Active/Inactive membership: Art, music or educational organization | Now I am going to read off a list of voluntary organizations. For each organization, could you tell me whether you are an active member, an inactive member or not a member of that type of organization?: Art, music or educational organization |
| V28 | Active/Inactive membership: Labor Union | Now I am going to read off a list of voluntary organizations. For each organization, could you tell me whether you are an active member, an inactive member or not a member of that type of organization?: Labor Union |
| V29 | Active/Inactive membership: Political party | Now I am going to read off a list of voluntary organizations. For each organization, could you tell me whether you are an active member, an inactive member or not a member of that type of organization?: Political party |
| V30 | Active/Inactive membership: Environmental organization | Now I am going to read off a list of voluntary organizations. For each organization, could you tell me whether you are an active member, an inactive member or not a member of that type of organization?: Environmental organization |
| V31 | Active/Inactive membership: Professional association | Now I am going to read off a list of voluntary organizations. For each organization, could you tell me whether you are an active member, an inactive member or not a member of that type of organization?: Professional association |
| V32 | Active/Inactive membership: Humanitarian or charitable organization | Now I am going to read off a list of voluntary organizations. For each organization, could you tell me whether you are an active member, an inactive member or not a member of that type of organization?: Humanitarian or charitable organization |
| V33 | Active/Inactive membership: Consumer organization | Now I am going to read off a list of voluntary organizations. For each organization, could you tell me whether you are an active member, an inactive member or not a member of that type of organization?: Consumer organization |
| V34 | Active/Inactive membership: Self-help group, mutual aid group | Now I am going to read off a list of voluntary organizations. For each organization, could you tell me whether you are an active member, an inactive member or not a member of that type of organization?: Self-help group, mutual aid group |
| V35 | Active/Inactive membership: Other organization | Now I am going to read off a list of voluntary organizations. For each organization, could you tell me whether you are an active member, an inactive member or not a member of that type of organization?: Other organization |
| V36 | Would not like to have as neighbors: Drug addicts | On this list are various groups of people. Could you please mention any that you would not like to have as neighbors?: Drug addicts |
| V37 | Would not like to have as neighbors: People of a different race | On this list are various groups of people. Could you please mention any that you would not like to have as neighbors?: People of a different race |
| **ID** | **LABEL** | **QUESTION** |
| V38 | Would not like to have as neighbors: People who have AIDS | On this list are various groups of people. Could you please mention any that you would not like to have as neighbors?: People who have AIDS |
| V39 | Would not like to have as neighbors: Immigrants/foreign workers | On this list are various groups of people. Could you please mention any that you would not like to have as neighbors?: Immigrants/Foreign workers |
| V40 | Would not like to have as neighbors: Homosexuals | On this list are various groups of people. Could you please mention any that you would not like to have as neighbors?: Homosexuals |
| V41 | Would not like to have as neighbors: People of a different religion | On this list are various groups of people. Could you please mention any that you would not like to have as neighbors?: People of a different religion |
| V42 | Would not like to have as neighbors: Heavy drinkers | On this list are various groups of people. Could you please mention any that you would not like to have as neighbors?: Heavy drinkers |
| V43 | Would not like to have as neighbors: Unmarried couples living together | On this list are various groups of people. Could you please mention any that you would not like to have as neighbors?: Unmarried couples living together |
| V44 | Would not like to have as neighbors: People who speak a different language | On this list are various groups of people. Could you please mention any that you would not like to have as neighbors?: People who speak a different language |
| V45 | When jobs are scarce, men should have more right to a job than women | Do you agree, disagree or neither agree nor disagree with the following statements?: "When jobs are scarce, men should have more right to a job than women" |
| V46 | When jobs are scarce, employers should give priority to people of this country over immigrants. | Do you agree, disagree or neither agree nor disagree with the following statements?: "When jobs are scarce, employers should give priority to people of this country over immigrants." |
| V47 | If a woman earns more money than her husband, it's almost certain to cause problems | Do you agree, disagree or neither agree nor disagree with the following statements?: "If a woman earns more money than her husband, it's almost certain to cause problems" |
| V48 | Having a job is the best way for a woman to be an independent person. | Do you agree, disagree or neither agree nor disagree with the following statements?: "Having a job is the best way for a woman to be an independent person." |
| V49 | One of my main goals in life has been to make my parents proud | For each of the following statements I read out, can you tell me how strongly you agree or disagree with each. Do you strongly agree, agree, disagree, or strongly disagree?: "One of my main goals in life has been to make my parents proud" |
| V50 | When a mother works for pay, the children suffer | For each of the following statements I read out, can you tell me how strongly you agree or disagree with each. Do you strongly agree, agree, disagree, or strongly disagree?: "When a mother works for pay, the children suffer" |
| V51 | On the whole, men make better political leaders than women do | For each of the following statements I read out, can you tell me how strongly you agree or disagree with each. Do you strongly agree, agree, disagree, or strongly disagree?: "On the whole, men make better political leaders than women do" |

| **ID** | **LABEL** | **QUESTION** |
| --- | --- | --- |
| V52 | A university education is more important for a boy than for a girl | For each of the following statements I read out, can you tell me how strongly you agree or disagree with each. Do you strongly agree, agree, disagree, or strongly disagree?: "A university education is more important for a boy than for a girl" |
| V53 | On the whole, men make better business executives than women do | For each of the following statements I read out, can you tell me how strongly you agree or disagree with each. Do you strongly agree, agree, disagree, or strongly disagree?: "On the whole, men make better business executives than women do" |
| V54 | Being a housewife is just as fulfilling as working for pay | For each of the following statements I read out, can you tell me how strongly you agree or disagree with each. Do you strongly agree, agree, disagree, or strongly disagree?: "Being a housewife is just as fulfilling as working for pay" |
| V55 | How much freedom of choice and control over own life | Some people feel they have completely free choice and control over their lives, while other people feel that what they do has no real effect on what happens to them. Please use this scale where 1 means "no choice at all" and 10 means "a great deal of choice" to indicate how much freedom of choice and control you feel you have over the way your life turns out: |
| V56 | Do you think most people would try to take advantage of you if they got a chance, or would they try to be fair? | Do you think most people would try to take advantage of you if they got a chance, or would they try to be fair? Please show your response on this card, where 1 means that “people would try to take advantage of you,” and 10 means that “people would try to be fair”: |
| V59 | Satisfaction with financial situation of household | How satisfied are you with the financial situation of your household? |
| V60 | Aims of country: first choice | People sometimes talk about what the aims of this country should be for the next ten years. On this card are listed some of the goals which different people would give top priority. Would you please say which one of these you, yourself, consider the most important? |
| V61 | Aims of country: second choice | And which would be the next most important? |
| V62 | Aims of respondent: first choice | If you had to choose, which one of the things on this card would you say is most important? |
| V63 | Aims of respondent: second choice | And which would be the next most important? |
| V64 | Most important: first choice | Here is another list. In your opinion, which one of these is most important? |
| V65 | Most important: second choice | And what would be the next most important? |
| V66 | Willingness to fight for your country | Of course, we all hope that there will not be another war, but if it were to come to that, would you be willing to fight for your country? |
| V67 | Future changes: Less importance placed on work in our lives | I'm going to read out a list of various changes in our way of life that might take place in the near future. Please tell me for each one, if it were to happen, whether you think it would be a good thing, a bad thing, or don't you mind? "Less importance placed on work in our lives" |
| V68 | Future changes: More emphasis on the development of technology | I'm going to read out a list of various changes in our way of life that might take place in the near future. Please tell me for each one, if it were to happen, whether you think it would be a good thing, a bad thing, or don't you mind?: "More emphasis on the development of technology" |
| V69 | Future changes: Greater respect for authority | I'm going to read out a list of various changes in our way of life that might take place in the near future. Please tell me for each one, if it were to happen, whether you think it would be a good thing, a bad thing, or don't you mind?: "Greater respect for authority" |
| **ID** | **LABEL** | **QUESTION** |
| V70 | Schwartz: It is important to this person to think up new ideas and be creative; to do things one’s own way | Now I will briefly describe some people. Using this card, would you please indicate for each description whether that person is very much like you, like you, somewhat like you, not like you, or not at all like you? "It is important to this person to think up new ideas and be creative; to do things one’s own way. " |
| V71 | Schwartz: It is important to this person to be rich; to have a lot of money and expensive things | Now I will briefly describe some people. Using this card, would you please indicate for each description whether that person is very much like you, like you, somewhat like you, not like you, or not at all like you? "It is important to this person to be rich; to have a lot of money and expensive things" |
| V72 | Schwartz: Living in secure surroundings is important to this person; to avoid anything that might be dangerous | Now I will briefly describe some people. Using this card, would you please indicate for each description whether that person is very much like you, like you, somewhat like you, not like you, or not at all like you? "Living in secure surroundings is important to this person; to avoid anything that might be dangerous" |
| V73 | Schwartz: It is important to this person to have a good time; to “spoil” oneself | Now I will briefly describe some people. Using this card, would you please indicate for each description whether that person is very much like you, like you, somewhat like you, not like you, or not at all like you? : "It is important to this person to have a good time; to “spoil” oneself" |
| V74 | Schwartz: It is important to this person to do something for the good of society | Now I will briefly describe some people. Using this card, would you please indicate for each description whether that person is very much like you, like you, somewhat like you, not like you, or not at all like you?: "It is important to this person to do something for the good of society" |
| V75 | Schwartz: Being very successful is important to this person; to have people recognize one’s achievements | Now I will briefly describe some people. Using this card, would you please indicate for each description whether that person is very much like you, like you, somewhat like you, not like you, or not at all like you? "Being very successful is important to this person; to have people recognize one’s achievements" |
| V76 | Schwartz: Adventure and taking risks are important to this person; to have an exciting life | Now I will briefly describe some people. Using this card, would you please indicate for each description whether that person is very much like you, like you, somewhat like you, not like you, or not at all like you? : "Adventure and taking risks are important to this person; to have an exciting life" |
| V77 | Schwartz: It is important to this person to always behave properly; to avoid doing anything people would say is wrong | Now I will briefly describe some people. Using this card, would you please indicate for each description whether that person is very much like you, like you, somewhat like you, not like you, or not at all like you?: "It is important to this person to always behave properly; to avoid doing anything people would say is wrong" |
| V78 | Schwartz: Looking after the environment is important to this person; to care for nature and save life resources | Now I will briefly describe some people. Using this card, would you please indicate for each description whether that person is very much like you, like you, somewhat like you, not like you, or not at all like you? : "Looking after the environment is important to this person; to care for nature and save life resources" |
| V79 | Schwartz: Tradition is important to this person; to follow the customs handed down by one’s religion or family | Now I will briefly describe some people. Using this card, would you please indicate for each description whether that person is very much like you, like you, somewhat like you, not like you, or not at all like you?: "Tradition is important to this person; to follow the customs handed down by one’s religion or family" |
| V80 | Most serious problem of the world | I’m going to read out some problems. Please indicate which of the following problems you consider the most serious one for the world as a whole? |
| V81 | Protecting environment vs. Economic growth | Here are two statements people sometimes make when discussing the environment and economic growth. Which of them comes closer to your own point of view?: |

| **ID** | **LABEL** | **QUESTION** |
| --- | --- | --- |
| V82 | Past two years: given money to ecological organization | During the past two years have you… Given money to an ecological organization? |
| V83 | Past two years: participated in demonstration for environment | During the past two years have you…Participated in a demonstration for some environmental cause? |
| V84 | Interest in politics | How interested would you say you are in politics? |
| V85 | Political action: Signing a petition | Now I’d like you to look at this card. I’m going to read out some forms of political action that people can take, and I’d like you to tell me, for each one, whether you have done any of these things, whether you might do it or would never under any circumstances do it: "Signing a petition" |
| V86 | Political action: Joining in boycotts | Now I’d like you to look at this card. I’m going to read out some forms of political action that people can take, and I’d like you to tell me, for each one, whether you have done any of these things, whether you might do it or would never under any circumstances do it: "Joining in boycotts" |
| V87 | Political action: Attending peaceful demonstrations | Now I’d like you to look at this card. I’m going to read out some forms of political action that people can take, and I’d like you to tell me, for each one, whether you have done any of these things, whether you might do it or would never under any circumstances do it: "Attending peaceful demonstrations" |
| V88 | Political action: Joining strikes | Now I’d like you to look at this card. I’m going to read out some forms of political action that people can take, and I’d like you to tell me, for each one, whether you have done any of these things, whether you might do it or would never under any circumstances do it: "Joining strikes" |
| V89 | Political action: Any other act of protest | Now I’d like you to look at this card. I’m going to read out some forms of political action that people can take, and I’d like you to tell me, for each one, whether you have done any of these things, whether you might do it or would never under any circumstances do it: "Any other act of protest" |
| V95 | Self positioning in political scale | In political matters, people talk of "the left" and "the right." How would you place your views on this scale, generally speaking? |
| V96 | Income equality | Now I'd like you to tell me your views on various issues. How would you place your views on this scale? 1 means you agree completely with the statement on the left; 10 means you agree completely with the statement on the right; and if your views fall somewhere in between, you can choose any number in between. "Incomes should be made more equal" vs. "We need larger income differences as incentives for individual effort" |
| V97 | Private vs state ownership of business | Now I'd like you to tell me your views on various issues. How would you place your views on this scale? 1 means you agree completely with the statement on the left; 10 means you agree completely with the statement on the right; and if your views fall somewhere in between, you can choose any number in between. "Private ownership of business and industry should be increased" vs. "Government ownership of business and industry should be increased" |
| V98 | Government responsibility | Now I'd like you to tell me your views on various issues. How would you place your views on this scale? 1 means you agree completely with the statement on the left; 10 means you agree completely with the statement on the right; and if your views fall somewhere in between, you can choose any number in between. "Government should take more responsibility to ensure that everyone is provided for" vs. "People should take more responsibility to provide for themselves" |
| **ID** | **LABEL** | **QUESTION** |
| V99 | Competition good or harmful | Now I'd like you to tell me your views on various issues. How would you place your views on this scale? 1 means you agree completely with the statement on the left; 10 means you agree completely with the statement on the right; and if your views fall somewhere in between, you can choose any number in between. "Competition is good. It stimulates people to work hard and develop new ideas" vs. "Competition is harmful. It brings out the worst in people" |
| V100 | Hard work brings success | Now I'd like you to tell me your views on various issues. How would you place your views on this scale? 1 means you agree completely with the statement on the left; 10 means you agree completely with the statement on the right; and if your views fall somewhere in between, you can choose any number in between. "In the long run, hard work usually brings a better life" vs. "Hard work doesn’t generally bring success—it’s more a matter of luck and connections" |
| V101 | Wealth accumulation | Now I'd like you to tell me your views on various issues. How would you place your views on this scale? 1 means you agree completely with the statement on the left; 10 means you agree completely with the statement on the right; and if your views fall somewhere in between, you can choose any number in between.: "People can only get rich at the expense of others" vs. "Wealth can grow so there’s enough for everyone" |
| V102 | How much you trust: Your family | I ‘d like to ask you how much you trust people from various groups. Could you tell me for each whether you trust people from this group completely, somewhat, not very much or not at all? Your family |
| V103 | How much you trust: Your neighborhood | I ‘d like to ask you how much you trust people from various groups. Could you tell me for each whether you trust people from this group completely, somewhat, not very much or not at all? Your neighborhood |
| V104 | How much you trust: People you know personally | I ‘d like to ask you how much you trust people from various groups. Could you tell me for each whether you trust people from this group completely, somewhat, not very much or not at all? People you know personally |
| V105 | How much you trust: People you meet for the first time | I ‘d like to ask you how much you trust people from various groups. Could you tell me for each whether you trust people from this group completely, somewhat, not very much or not at all? People you meet for the first time |
| V106 | How much you trust: People of another religion | I ‘d like to ask you how much you trust people from various groups. Could you tell me for each whether you trust people from this group completely, somewhat, not very much or not at all? : People of another religion |
| V107 | How much you trust: People of another nationality | I ‘d like to ask you how much you trust people from various groups. Could you tell me for each whether you trust people from this group completely, somewhat, not very much or not at all? People of another nationality |
| V108 | Confidence: The Churches | I am going to name a number of organizations. For each one, could you tell me how much confidence you have in them: is it a great deal of confidence, quite a lot of confidence, not very much confidence or none at all? The churches (*) * [Substitute “religious organizations” in non-Christian countries; “the Church” in Catholic countries] |
| V109 | Confidence: The armed forces | I am going to name a number of organizations. For each one, could you tell me how much confidence you have in them: is it a great deal of confidence, quite a lot of confidence, not very much confidence or none at all?: The armed forces |
| V110 | Confidence: The press | I am going to name a number of organizations. For each one, could you tell me how much confidence you have in them: is it a great deal of confidence, quite a lot of confidence, not very much confidence or none at all?: The press |

| **ID** | **LABEL** | **QUESTION** |
| --- | --- | --- |
| V111 | Confidence: Television | I am going to name a number of organizations. For each one, could you tell me how much confidence you have in them: is it a great deal of confidence, quite a lot of confidence, not very much confidence or none at all?: Television |
| V112 | Confidence: Labour Unions | I am going to name a number of organizations. For each one, could you tell me how much confidence you have in them: is it a great deal of confidence, quite a lot of confidence, not very much confidence or none at all?: Labor unions |
| V113 | Confidence: The police | I am going to name a number of organizations. For each one, could you tell me how much confidence you have in them: is it a great deal of confidence, quite a lot of confidence, not very much confidence or none at all?: The police |
| V114 | Confidence: The courts | I am going to name a number of organizations. For each one, could you tell me how much confidence you have in them: is it a great deal of confidence, quite a lot of confidence, not very much confidence or none at all?: The courts |
| V115 | Confidence: The government (in your nation’s capital) | I am going to name a number of organizations. For each one, could you tell me how much confidence you have in them: is it a great deal of confidence, quite a lot of confidence, not very much confidence or none at all?: The government (in your nation’s capital) |
| V116 | Confidence: Political Parties | I am going to name a number of organizations. For each one, could you tell me how much confidence you have in them: is it a great deal of confidence, quite a lot of confidence, not very much confidence or none at all?: Political parties |
| V117 | Confidence: Parliament | I am going to name a number of organizations. For each one, could you tell me how much confidence you have in them: is it a great deal of confidence, quite a lot of confidence, not very much confidence or none at all?: Parliament |
| V118 | Confidence: The Civil service | I am going to name a number of organizations. For each one, could you tell me how much confidence you have in them: is it a great deal of confidence, quite a lot of confidence, not very much confidence or none at all? The Civil service |
| V119 | Confidence: Universities | I am going to name a number of organizations. For each one, could you tell me how much confidence you have in them: is it a great deal of confidence, quite a lot of confidence, not very much confidence or none at all?: Universities |
| V120 | Confidence: Major Companies | I am going to name a number of organizations. For each one, could you tell me how much confidence you have in them: is it a great deal of confidence, quite a lot of confidence, not very much confidence or none at all?: Major Companies |
| V121 | Confidence: Banks | I am going to name a number of organizations. For each one, could you tell me how much confidence you have in them: is it a great deal of confidence, quite a lot of confidence, not very much confidence or none at all?: Banks |
| V122 | Confidence: Environmental organizations | I am going to name a number of organizations. For each one, could you tell me how much confidence you have in them: is it a great deal of confidence, quite a lot of confidence, not very much confidence or none at all?: Environmental organizations |
| V123 | Confidence: Women's organizations | I am going to name a number of organizations. For each one, could you tell me how much confidence you have in them: is it a great deal of confidence, quite a lot of confidence, not very much confidence or none at all? Women’s organizations |
| V124 | Confidence: Charitable or humanitarian organizations | I am going to name a number of organizations. For each one, could you tell me how much confidence you have in them: is it a great deal of confidence, quite a lot of confidence, not very much confidence or none at all? Charitable or humanitarian organizations |
| V126 | Confidence: The United Nations | I am going to name a number of organizations. For each one, could you tell me how much confidence you have in them: is it a great deal of confidence, quite a lot of confidence, not very much confidence or none at all?: The United Nations |

| **ID** | **LABEL** | **QUESTION** |
| --- | --- | --- |
| V127 | Political system: Having a strong leader who does not have to bother with parliament and elections | I'm going to describe various types of political systems and ask what you think about each as a way of governing this country. For each one, would you say it is a very good, fairly good, fairly bad or very bad way of governing this country? Having a strong leader who does not have to bother with parliament and elections |
| V128 | Political system: Having experts, not government, make decisions according to what they think is best for the country | I'm going to describe various types of political systems and ask what you think about each as a way of governing this country. For each one, would you say it is a very good, fairly good, fairly bad or very bad way of governing this country? Having experts, not government, make decisions according to what they think is best for the country |
| V129 | Political system: Having the army rule | I'm going to describe various types of political systems and ask what you think about each as a way of governing this country. For each one, would you say it is a very good, fairly good, fairly bad or very bad way of governing this country? Having the army rule |
| V130 | Political system: Having a democratic political system | I'm going to describe various types of political systems and ask what you think about each as a way of governing this country. For each one, would you say it is a very good, fairly good, fairly bad or very bad way of governing this country? Having a democratic political system |
| V131 | Democracy: Governments tax the rich and subsidize the poor. | Many things are desirable, but not all of them are essential characteristics of democracy. Please tell me for each of the following things how essential you think it is as a characteristic of democracy. Use this scale where 1 means “not at all an essential characteristic of democracy” and 10 means it definitely is “an essential characteristic of democracy”: Governments tax the rich and subsidize the poor |
| V132 | Democracy: Religious authorities interpret the laws. | Many things are desirable, but not all of them are essential characteristics of democracy. Please tell me for each of the following things how essential you think it is as a characteristic of democracy. Use this scale where 1 means “not at all an essential characteristic of democracy” and 10 means it definitely is “an essential characteristic of democracy”: Religious authorities ultimately interpret the laws |
| V134 | Democracy: People receive state aid for unemployment. | Many things are desirable, but not all of them are essential characteristics of democracy. Please tell me for each of the following things how essential you think it is as a characteristic of democracy. Use this scale where 1 means “not at all an essential characteristic of democracy” and 10 means it definitely is “an essential characteristic of democracy”: People receive state aid for unemployment |
| V135 | Democracy: The army takes over when government is incompetent. | Many things are desirable, but not all of them are essential characteristics of democracy. Please tell me for each of the following things how essential you think it is as a characteristic of democracy. Use this scale where 1 means “not at all an essential characteristic of democracy” and 10 means it definitely is “an essential characteristic of democracy”: The army takes over when government is incompetent |
| V136 | Democracy: Civil rights protect people’s liberty from state oppression | Many things are desirable, but not all of them are essential characteristics of democracy. Please tell me for each of the following things how essential you think it is as a characteristic of democracy. Use this scale where 1 means “not at all an essential characteristic of democracy” and 10 means it definitely is “an essential characteristic of democracy”: Civil rights protect people from state oppression |

| **ID** | **LABEL** | **QUESTION** |
| --- | --- | --- |
| V137 | Democracy: The state makes people's incomes equal | Many things are desirable, but not all of them are essential characteristics of democracy. Please tell me for each of the following things how essential you think it is as a characteristic of democracy. Use this scale where 1 means “not at all an essential characteristic of democracy” and 10 means it definitely is “an essential characteristic of democracy”: The state makes people’s incomes equal |
| V138 | Democracy: People obey their rulers | Many things are desirable, but not all of them are essential characteristics of democracy. Please tell me for each of the following things how essential you think it is as a characteristic of democracy. Use this scale where 1 means “not at all an essential characteristic of democracy” and 10 means it definitely is “an essential characteristic of democracy”: People obey their rulers |
| V141 | How democratically is this country being governed today | And how democratically is this country being governed today? Again using a scale from 1 to 10, where 1 means that it is “not at all democratic” and 10 means that it is “completely democratic,” what position would you choose? |
| V142 | How much respect is there for individual human rights nowadays in this country | How much respect is there for individual human rights nowadays in this country?. Do you feel there is: |
| V143 | Thinking about meaning and purpose of life | Now let’s turn to another topic. How often, if at all, do you think about the meaning and purpose of life? |
| V145 | How often do you attend religious services | Apart from weddings and funerals, about how often do you attend religious services these days? |
| V146 | How often to you pray | Apart from weddings and funerals, about how often do you pray? |
| V147 | Religious person | Independently of whether you attend religious services or not, would you say you are: |
| V148 | Believe in: God | Do you believe in God? |
| V149 | Believe in: hell | Do you believe in hell? |
| V150 | Meaning of religion: To follow religious norms and ceremonies vs To do good to other people | With which one of the following statements do you agree most? The basic meaning of religion is: "To follow religious norms and ceremonies" or "To do good to other people" |
| V151 | Meaning of religion: To make sense of life after death vs To make sense of life in this world | And with which of the following statements do you agree most? The basic meaning of religion is: "To make sense of life after death" or "To make sense of life in this world" |
| V153 | Whenever science and religion conflict, religion is always right | Please tell us if you strongly agree, agree, disagree, or strongly disagree with the following statements: "Whenever science and religion conflict, religion is always right" |
| V154 | The only acceptable religion is my religion | Please tell us if you strongly agree, agree, disagree, or strongly disagree with the following statements: "The only acceptable religion is my religion" |
| V155 | All religions should be taught in public schools | Please tell us if you strongly agree, agree, disagree, or strongly disagree with the following statements: All religions should be taught in our public schools |
| V156 | People who belong to different religions are probably just as moral as those who belong to mine | Please tell us if you strongly agree, agree, disagree, or strongly disagree with the following statements: People who belong to different religions are probably just as moral as those who belong to mine |
| V157 | Social position: People in their 20s | I’m interested in how you think most people in this country view the position in society of people in their 20s, people in their 40s and people over 70.* Using this card, please tell me where most people would place the social position of … …people in their 20’s? |

| **ID** | **LABEL** | **QUESTION** |
| --- | --- | --- |
| V158 | Social position: People in their 40s | I’m interested in how you think most people in this country view the position in society of people in their 20s, people in their 40s and people over 70.* Using this card, please tell me where most people would place the social position of … …people in their 40’s? |
| V159 | Social position: People in their 70s | I’m interested in how you think most people in this country view the position in society of people in their 20s, people in their 40s and people over 70.* Using this card, please tell me where most people would place the social position of ……people over 70? |
| V160 | Is a 30-year old boss acceptable | Please tell me how acceptable or unacceptable you think most people in [country] would find it if a suitably qualified 30 year old was appointed as their boss? |
| V161 | People over 70: are seen as friendly | Now think about those aged over 70*. Using the same card please tell me how likely it is that most people in [country] view those over 70… …as friendly? |
| V162 | People over 70: are seen as competent | Now think about those aged over 70*. Using the same card please tell me how likely it is that most people in [country] view those over 70… …as competent? |
| V163 | People over 70: viewed with respect | Now think about those aged over 70*. Using the same card please tell me how likely it is that most people in [country] view those over 70… …with respect? |
| V164 | Is a 70-year old boss acceptable | Please tell me how acceptable or unacceptable you think most people in [country] would find it if a suitably qualified 70* year old was appointed as their boss? |
| V165 | Older people are not respected much these days | Now could you tell me whether you agree, agree strongly, disagree or disagree strongly with each of the following statements? Older people are not respected much these days |
| V166 | Older people get more than their fair share from the government | Now could you tell me whether you agree, agree strongly, disagree or disagree strongly with each of the following statements? Older people get more than their fair share from the government |
| V167 | Older people are a burden on society | Now could you tell me whether you agree, agree strongly, disagree or disagree strongly with each of the following statements? Older people are a burden on society. |
| V168 | Companies that employ young people perform better than those that employ people of different ages | Now could you tell me whether you agree, agree strongly, disagree or disagree strongly with each of the following statements? Companies that employ young people perform better than those that employ people of different ages. |
| V169 | Old people have too much political influence | Now could you tell me whether you agree, agree strongly, disagree or disagree strongly with each of the following statements? Old people have too much political influence. |
| V170 | Secure in neighborhood | Could you tell me how secure do you feel these days in your neighborhood? |
| V176 | Things done for reasons of security: Didn’t carry much money | Which of the following things have you done for reasons of security? Didn’t carry much money |
| V177 | Things done for reasons of security: Preferred not to go out at night | Which of the following things have you done for reasons of security? Preferred not to go out at night |
| V178 | Things done for reasons of security: Carried a knife, gun or other weapon | Which of the following things have you done for reasons of security? Carried a knife, gun or other weapon |
| V181 | Worries: Losing my job or not finding a job | To what degree are you worried about the following situations? Losing my job or not finding a job |

| **ID** | **LABEL** | **QUESTION** |
| --- | --- | --- |
| V182 | Worries: Not being able to give one's children a good education | To what degree are you worried about the following situations? Not being able to give my children a good education |
| V183 | Worries: A war involving my country | To what degree are you worried about the following situations? A war involving my country |
| V184 | Worries: A terrorist attack | To what degree are you worried about the following situations? A terrorist attack |
| V185 | Worries: A civil war | To what degree are you worried about the following situations? A civil war |
| V186 | Worries: Government wire-tapping or reading my mail or email | To what degree are you worried about the following situations? Government wire-tapping or reading my mail or email |
| V187 | Under some conditions, war is necessary to obtain justice | Do you agree or disagree with the following statement: “Under some conditions, war is necessary to obtain justice” |
| V192 | Science and technology are making our lives healthier, easier, and more comfortable | Now, I would like to read some statements and ask how much you agree or disagree with each of these statements. For these questions, a 1 means that you “completely disagree” and a 10 means that you “completely agree”: "Science and technology are making our lives healthier, easier, and more comfortable" |
| V194 | We depend too much on science and not enough on faith | Now, I would like to read some statements and ask how much you agree or disagree with each of these statements. For these questions, a 1 means that you “completely disagree” and a 10 means that you “completely agree”: We depend too much on science and not enough on faith |
| V195 | One of the bad effects of science is that it breaks down people’s ideas of right and wrong | Now, I would like to read some statements and ask how much you agree or disagree with each of these statements. For these questions, a 1 means that you “completely disagree” and a 10 means that you “completely agree”: One of the bad effects of science is that it breaks down people’s ideas of right and wrong |
| V196 | It is not important for me to know about science in my daily life | Now, I would like to read some statements and ask how much you agree or disagree with each of these statements. For these questions, a 1 means that you “completely disagree” and a 10 means that you “completely agree”: It is not important for me to know about science in my daily life |
| V197 | The world is better off, or worse off, because of science and technology | All things considered, would you say that the world is better off, or worse off, because of science and technology?. Please tell me which comes closest to your view on this scale: 1 means that “the world is a lot worse off,” and 10 means that “the world is a lot better off”. |
| V198 | Justifiable: Claiming government benefits to which you are not entitled | Please tell me for each of the following actions whether you think it can always be justified, never be justified, or something in between: Claiming government benefits to which you are not entitled |
| V199 | Justifiable: Avoiding a fare on public transport | Please tell me for each of the following actions whether you think it can always be justified, never be justified, or something in between: Avoiding a fare on public transport |
| V200 | Justifiable: Stealing property | Please tell me for each of the following actions whether you think it can always be justified, never be justified, or something in between: Stealing property |
| V201 | Justifiable: Cheating on taxes if you have a chance | Please tell me for each of the following actions whether you think it can always be justified, never be justified, or something in between: Cheating on taxes if you have a chance |
| V202 | Justifiable: Someone accepting a bribe in the course of their duties | Please tell me for each of the following actions whether you think it can always be justified, never be justified, or something in between: Someone accepting a bribe in the course of their duties |

| **ID** | **LABEL** | **QUESTION** |
| --- | --- | --- |
| V203 | Justifiable: Homosexuality | Please tell me for each of the following actions whether you think it can always be justified, never be justified, or something in between: Homosexuality |
| V204 | Justifiable: Abortion | Please tell me for each of the following actions whether you think it can always be justified, never be justified, or something in between: Abortion |
| V205 | Justifiable: Divorce | Please tell me for each of the following actions whether you think it can always be justified, never be justified, or something in between: Divorce |
| V206 | Justifiable: Sex before marriage | Please tell me for each of the following actions whether you think it can always be justified, never be justified, or something in between: Sex before marriage |
| V207 | Justifiable: Suicide | Please tell me for each of the following actions whether you think it can always be justified, never be justified, or something in between: Suicide |
| V208 | Justifiable: For a man to beat his wife | Please tell me for each of the following actions whether you think it can always be justified, never be justified, or something in between: For a man to beat his wife |
| V209 | Justifiable: Parents beating children | Please tell me for each of the following actions whether you think it can always be justified, never be justified, or something in between: Parents beating children |
| V210 | Justifiable: Violence against other people | Please tell me for each of the following actions whether you think it can always be justified, never be justified, or something in between: Violence against other people |
| V211 | How proud of nationality | How proud are you to be [Nationality]*?: * [Substitute your own nationality] |
| V212 | I see myself as a world citizen | People have different views about themselves and how they relate to the world. Using this card, would you tell me how strongly you agree or disagree with each of the following statements about how you see yourself? I see myself as a world citizen |
| V213 | I see myself as part of my local community | People have different views about themselves and how they relate to the world. Using this card, would you tell me how strongly you agree or disagree with each of the following statements about how you see yourself? I see myself as part of my local community |
| V214 | I see myself as part of the [country] nation | People have different views about themselves and how they relate to the world. Using this card, would you tell me how strongly you agree or disagree with each of the following statements about how you see yourself? I see myself as part of the [French]* nation * [Substitute your country’s nationality for “French”] |
| V216 | I see myself as an autonomous individual | People have different views about themselves and how they relate to the world. Using this card, would you tell me how strongly you agree or disagree with each of the following statements about how you see yourself? I see myself as an autonomous individual |
| V217 | Information source: Daily newspaper | People learn what is going on in this country and the world from various sources. For each of the following sources, please indicate whether you use it to obtain information daily, weekly, monthly, less than monthly or never: daily newspaper |
| V218 | Information source: Printed magazines | People learn what is going on in this country and the world from various sources. For each of the following sources, please indicate whether you use it to obtain information daily, weekly, monthly, less than monthly or never: Printed magazines |
| V219 | Information source: TV news | People learn what is going on in this country and the world from various sources. For each of the following sources, please indicate whether you use it to obtain information daily, weekly, monthly, less than monthly or never: TV news |

| **ID** | **LABEL** | **QUESTION** |
| --- | --- | --- |
| V220 | Information source: Radio news | People learn what is going on in this country and the world from various sources. For each of the following sources, please indicate whether you use it to obtain information daily, weekly, monthly, less than monthly or never: Radio news |
| V221 | Information source: Mobile phone | People learn what is going on in this country and the world from various sources. For each of the following sources, please indicate whether you use it to obtain information daily, weekly, monthly, less than monthly or never: Mobile phone |
| V222 | Information source: Email | People learn what is going on in this country and the world from various sources. For each of the following sources, please indicate whether you use it to obtain information daily, weekly, monthly, less than monthly or never: Email |
| V223 | Information source: Internet | People learn what is going on in this country and the world from various sources. For each of the following sources, please indicate whether you use it to obtain information daily, weekly, monthly, less than monthly or never: Internet |
| V224 | Information source: Talk with friends or colleagues | People learn what is going on in this country and the world from various sources. For each of the following sources, please indicate whether you use it to obtain information daily, weekly, monthly, less than monthly or never: Talk with friends or colleagues |
| V225 | How often use of a personal computer | How often, if ever, do you use a personal computer? |
| V226 | Vote in elections: local level | When elections take place, do you vote always, usually or never?: Local level |
| V227 | Vote in elections: National level | When elections take place, do you vote always, usually or never?: National level |
