# Supplementary material for: Religiosity, neutrality, fairness, skepticism, and societal tranquility: A data science analysis of the World Values Survey
Source: PLoS One. 2021 Jan 11;16(1):e0245231. doi: 10.1371/journal.pone.0245231 (PMC7799817; doi:10.1371/journal.pone.0245231)
Supplement: S2 Table — (DOCX) [file pone.0245231.s002.docx]

# S2 Table - WVS questions included in confirmatory factor analysis (CFA) and factor loadings

| **VAR** | **QUESTION** | **F1** | **F2** | **F3** | **F4** | **F5** | **Interpretation** |
| --- | --- | --- | --- | --- | --- | --- | --- |
| V9 | For each of the following, indicate how important it is in your life. Would you say it is: Religion | **-0.61** | 0.00 | 0.00 | 0.00 | 0.00 | Religion is very important |
| V148 | Do you believe in God? | **-0.61** | 0.00 | 0.00 | 0.00 | 0.00 | Believe in God |
| V153 | Please tell us if you strongly agree, agree, disagree, or strongly disagree with the following statements:  "Whenever science and religion conflict, religion is always right" | **-0.51** | 0.00 | 0.00 | 0.00 | 0.00 | Strongly agree that whenever science and religion conflict, religion is always right. |
| V146 | Apart from weddings and funerals, about how often do you pray? | **-0.47** | 0.00 | 0.00 | 0.00 | 0.00 | You pray several times a day |
| V19 | Here is a list of qualities that children can be encouraged to learn at home. Which, if any, do you consider to be especially important?: Religious faith | **-0.44** | 0.00 | 0.00 | 0.00 | 0.00 | Religious faith is an important quality for children to learn at home. |
| V149 | Do you believe in hell? | **-0.43** | 0.00 | 0.00 | 0.00 | 0.00 | Believe in hell |
| V147 | Independently of whether you attend religious services or not, would you say you are: | **-0.41** | 0.00 | 0.00 | 0.00 | 0.00 | You are a religious person |
| V206 | Please tell me for each of the following actions whether you think it can always be justified, never be justified, or something in between: Sex before marriage | **-0.41** | 0.00 | 0.00 | 0.00 | 0.00 | Sex before marriage is never justifiable |
| V154 | Please tell us if you strongly agree, agree, disagree, or strongly disagree with the following statements: "The only acceptable religion is my religion" | **-0.38** | 0.00 | 0.00 | 0.00 | 0.00 | You strongly agree that the only acceptable religion is my religion |
| V203 | Please tell me for each of the following actions whether you think it can always be justified, never be justified, or something in between: Homosexuality | **-0.37** | **-0.23** | 0.00 | 0.00 | 0.00 | Homosexuality is never justifiable. |
| V204 | Please tell me for each of the following actions whether you think it can always be justified, never be justified, or something in between: Abortion | **-0.34** | 0.00 | 0.00 | 0.00 | 0.00 | Abortion is never justifiable |

| **VAR** | **QUESTION** | | | **F1** | | **F2** | | **F3** | | **F4** | | **F5** | | **Interpretation** |  |
| --- | --- | --- | --- | --- | --- | --- | --- | --- | --- | --- | --- | --- | --- | --- | --- |
| V33 | Now I am going to read off a list of voluntary organizations. For each organization, could you tell me whether you are an active member, an inactive member or not a member of that type of organization?: Consumer organization | | | 0.00 | | **-0.76** | | 0.00 | | 0.00 | | 0.00 | | Not a member of a consumer organization |  |
| V30 | Now I am going to read off a list of voluntary organizations. For each organization, could you tell me whether you are an active member, an inactive member or not a member of that type of organization?: Environmental organization | | | 0.00 | | **-0.76** | | 0.00 | | 0.00 | | 0.00 | | Not a member of an environmental organization |  |
| V32 | Now I am going to read off a list of voluntary organizations. For each organization, could you tell me whether you are an active member, an inactive member or not a member of that type of organization?: Humanitarian or charitable organization | | | 0.00 | | **-0.74** | | 0.00 | | 0.00 | | 0.00 | | Not a member of a humanitarian or charitable organization |  |
| V34 | Now I am going to read off a list of voluntary organizations. For each organization, could you tell me whether you are an active member, an inactive member or not a member of that type of organization?: Self-help group, mutual aid group | | | 0.00 | | **-0.72** | | 0.00 | | 0.00 | | 0.00 | | Not a member of a self help or mutual aid group |  |
| V31 | Now I am going to read off a list of voluntary organizations. For each organization, could you tell me whether you are an active member, an inactive member or not a member of that type of organization?: Professional association | | | 0.00 | | **-0.71** | | 0.00 | | 0.00 | | 0.00 | | Not a member of a professional association |  |
| V27 | Now I am going to read off a list of voluntary organizations. For each organization, could you tell me whether you are an active member, an inactive member or not a member of that type of organization?: Art, music or educational organization | | | 0.00 | | **-0.65** | | 0.00 | | 0.00 | | 0.00 | | Not a member of an art, music, or education organization |  |
| V35 | Now I am going to read off a list of voluntary organizations. For each organization, could you tell me whether you are an active member, an inactive member or not a member of that type of organization?: Other organization | | | 0.00 | | **-0.65** | | 0.00 | | 0.00 | | 0.00 | | Not a member of other organizations |  |
| V29 | Now I am going to read off a list of voluntary organizations. For each organization, could you tell me whether you are an active member, an inactive member or not a member of that type of organization?: Political party | | | 0.00 | | **-0.61** | | 0.00 | | 0.00 | | 0.00 | | Not a member of political party |  |
| V26 | Now I am going to read off a list of voluntary organizations. For each organization, could you tell me whether you are an active member, an inactive member or not a member of that type of organization?: Sport or recreational organization | | | 0.00 | | **-0.60** | | 0.00 | | 0.00 | | 0.00 | | Not a member of a sport or recreational organization |  |
| **VAR** | | **QUESTION** | **F1** | | **F2** | | **F3** | | **F4** | | **F5** | | **Interpretation** | | |
| V25 | | Now I am going to read off a list of voluntary organizations. For each organization, could you tell me whether you are an active member, an inactive member or not a member of that type of organization?: Church or religious organization | 0.00 | | **-0.60** | | 0.00 | | 0.00 | | 0.00 | | Not a member of a church or religious organization | | |
| V28 | | Now I am going to read off a list of voluntary organizations. For each organization, could you tell me whether you are an active member, an inactive member or not a member of that type of organization?: Labor Union | 0.00 | | **-0.59** | | 0.00 | | 0.00 | | 0.00 | | Not a member of a labor union | | |
| V85 | | Now I’d like you to look at this card. I’m going to read out some forms of political action that people can take, and I’d like you to tell me, for each one, whether you have done any of these things, whether you might do it or would never under any circumstances do it: "Signing a petition" | 0.00 | | **0.25** | | 0.00 | | 0.00 | | 0.00 | | Would never sign a petition | | |
| V83 | | During the past two years have you…Participated in a demonstration for some environmental cause? | 0.00 | | **0.32** | | 0.00 | | 0.00 | | 0.00 | | No participation in a demonstration for some environmental cause | | |
| V200 | | Please tell me for each of the following actions whether you think it can always be justified, never be justified, or something in between: Stealing property | 0.00 | | 0.00 | | **-0.77** | | 0.00 | | 0.00 | | Never justifiable to steal property | | |
| V202 | | Please tell me for each of the following actions whether you think it can always be justified, never be justified, or something in between: Someone accepting a bribe in the course of their duties | 0.00 | | 0.00 | | **-0.74** | | 0.00 | | 0.00 | | Never justifiable for someone to accept a bribe in the course of their duties | | |
| V201 | | Please tell me for each of the following actions whether you think it can always be justified, never be justified, or something in between: Cheating on taxes if you have a chance | 0.00 | | 0.00 | | **-0.70** | | 0.00 | | 0.00 | | Cheating on taxes in you have the chance is never justifiable | | |
| V210 | | Please tell me for each of the following actions whether you think it can always be justified, never be justified, or something in between: Violence against other people | 0.00 | | 0.00 | | **-0.63** | | 0.00 | | 0.00 | | Violence against other people is never justifiable | | |
| V208 | | Please tell me for each of the following actions whether you think it can always be justified, never be justified, or something in between: For a man to beat his wife | 0.00 | | 0.00 | | **-0.58** | | 0.00 | | 0.00 | | A man to beat his wife is never justifiable. | | |
| V199 | | Please tell me for each of the following actions whether you think it can always be justified, never be justified, or something in between: Avoiding a fare on public transport | 0.00 | | 0.00 | | **-0.58** | | 0.00 | | 0.00 | | Avoiding fare on public transport is never justifiable | | |

| **VAR** | **QUESTION** | **F1** | **F2** | **F3** | **F4** | **F5** | **Interpretation** |
| --- | --- | --- | --- | --- | --- | --- | --- |
| V198 | Please tell me for each of the following actions whether you think it can always be justified, never be justified, or something in between: Claiming government benefits to which you are not entitled | 0.00 | 0.00 | **-0.51** | 0.00 | 0.00 | Claiming government benefits to which you are not entitle is never justifiable |
| V117 | I am going to name a number of organizations. For each one, could you tell me how much confidence you have in them: is it a great deal of confidence, quite a lot of confidence, not very much confidence or none at all?: Parliament | 0.00 | 0.00 | 0.00 | **0.65** | 0.00 | No confidence at all in Parliament |
| V118 | I am going to name a number of organizations. For each one, could you tell me how much confidence you have in them: is it a great deal of confidence, quite a lot of confidence, not very much confidence or none at all? The Civil service | 0.00 | 0.00 | 0.00 | **0.59** | 0.00 | No confidence at all in the Civil Service |
| V115 | I am going to name a number of organizations. For each one, could you tell me how much confidence you have in them: is it a great deal of confidence, quite a lot of confidence, not very much confidence or none at all?: The government (in your nation’s capital) | 0.00 | 0.00 | 0.00 | **0.59** | 0.00 | No confidence at all in the government (in your nation's capital) |
| V116 | I am going to name a number of organizations. For each one, could you tell me how much confidence you have in them: is it a great deal of confidence, quite a lot of confidence, not very much confidence or none at all?: Political parties | 0.00 | 0.00 | 0.00 | **0.58** | 0.00 | No confidence at all in political parties |
| V114 | I am going to name a number of organizations. For each one, could you tell me how much confidence you have in them: is it a great deal of confidence, quite a lot of confidence, not very much confidence or none at all?: The courts | 0.00 | 0.00 | 0.00 | **0.54** | 0.00 | No confidence at all in the courts |
| V112 | I am going to name a number of organizations. For each one, could you tell me how much confidence you have in them: is it a great deal of confidence, quite a lot of confidence, not very much confidence or none at all?: Labor unions | 0.00 | 0.00 | 0.00 | **0.50** | 0.00 | No confidence at all in labor unions |
| V110 | I am going to name a number of organizations. For each one, could you tell me how much confidence you have in them: is it a great deal of confidence, quite a lot of confidence, not very much confidence or none at all?: The press | 0.00 | 0.00 | 0.00 | **0.48** | 0.00 | No confidence at all in the press |
| V111 | I am going to name a number of organizations. For each one, could you tell me how much confidence you have in them: is it a great deal of confidence, quite a lot of confidence, not very much confidence or none at all?: Television | 0.00 | 0.00 | 0.00 | **0.47** | 0.00 | No confidence at all in television |

| **VAR** | | **QUESTION** | **F1** | | | **F2** | | **F3** | | **F4** | | **F5** | | **Interpretation** | |
| --- | --- | --- | --- | --- | --- | --- | --- | --- | --- | --- | --- | --- | --- | --- | --- |
| V120 | I am going to name a number of organizations. For each one, could you tell me how much confidence you have in them: is it a great deal of confidence, quite a lot of confidence, not very much confidence or none at all?: Major Companies | | | 0.00 | 0.00 | | 0.00 | | **0.47** | | 0.00 | | No confidence at all in major companies | |  |
| V113 | I am going to name a number of organizations. For each one, could you tell me how much confidence you have in them: is it a great deal of confidence, quite a lot of confidence, not very much confidence or none at all?: The police | | | 0.00 | 0.00 | | 0.00 | | **0.46** | | 0.00 | | No confidence at all in the police | |  |
| V121 | I am going to name a number of organizations. For each one, could you tell me how much confidence you have in them: is it a great deal of confidence, quite a lot of confidence, not very much confidence or none at all?: Banks | | | 0.00 | 0.00 | | 0.00 | | **0.46** | | 0.00 | | No confidence at all in banks | |  |
| V122 | I am going to name a number of organizations. For each one, could you tell me how much confidence you have in them: is it a great deal of confidence, quite a lot of confidence, not very much confidence or none at all?: Environmental organizations | | | 0.00 | 0.00 | | 0.00 | | **0.45** | | 0.00 | | No confidence at all in environmental organizations | |  |
| V123 | I am going to name a number of organizations. For each one, could you tell me how much confidence you have in them: is it a great deal of confidence, quite a lot of confidence, not very much confidence or none at all? Women’s organizations | | | 0.00 | 0.00 | | 0.00 | | **0.45** | | 0.00 | | No confidence at all in women's organizations | |  |
| V126 | I am going to name a number of organizations. For each one, could you tell me how much confidence you have in them: is it a great deal of confidence, quite a lot of confidence, not very much confidence or none at all?: The United Nations | | | 0.00 | 0.00 | | 0.00 | | **0.44** | | 0.00 | | No confidence at all in the United Nations | |  |
| V119 | I am going to name a number of organizations. For each one, could you tell me how much confidence you have in them: is it a great deal of confidence, quite a lot of confidence, not very much confidence or none at all?: Universities | | | 0.00 | 0.00 | | 0.00 | | **0.44** | | 0.00 | | No confidence at all in Universities | |  |
| V124 | I am going to name a number of organizations. For each one, could you tell me how much confidence you have in them: is it a great deal of confidence, quite a lot of confidence, not very much confidence or none at all? Charitable or humanitarian organizations | | | 0.00 | 0.00 | | 0.00 | | **0.41** | | 0.00 | | No confidence at all in charitable or humanitarian organizations | |  |
| V109 | I am going to name a number of organizations. For each one, could you tell me how much confidence you have in them: is it a great deal of confidence, quite a lot of confidence, not very much confidence or none at all?: The armed forces | | | 0.00 | 0.00 | | 0.00 | | **0.38** | | 0.00 | | No confidence at all in the armed forces | |  |
| V142 | How much respect is there for individual human rights nowadays in this country?. Do you feel there is: | | | 0.00 | 0.00 | | 0.00 | | **0.25** | | 0.00 | | There is no respect at all for individual human rights nowadays in this country | |  |

| **VAR** | **QUESTION** | **F1** | **F2** | | **F3** | | **F4** | | **F5** | | **Interpretation** | |  |
| --- | --- | --- | --- | --- | --- | --- | --- | --- | --- | --- | --- | --- | --- |
| V185 | To what degree are you worried about the following situations? A civil war | 0.00 | | 0.00 | | 0.00 | | 0.00 | | **0.82** | | No at all worried about a civil war | |
| V183 | To what degree are you worried about the following situations? A war involving my country | 0.00 | | 0.00 | | 0.00 | | 0.00 | | **0.80** | | Not at all worried about a war involving my country | |
| V184 | To what degree are you worried about the following situations? A terrorist attack | 0.00 | | 0.00 | | 0.00 | | 0.00 | | **0.80** | | Not at all worried about a terrorist attack | |
| V182 | To what degree are you worried about the following situations? Not being able to give my children a good education | 0.00 | | 0.00 | | 0.00 | | 0.00 | | **0.49** | | Not at all worried about not being able to give my children a good education | |
| V186 | To what degree are you worried about the following situations? Government wire-tapping or reading my mail or email | 0.00 | | 0.00 | | 0.00 | | 0.00 | | **0.47** | | Not at all worried about the government wire-tapping or reading my mail or email | |
| V181 | To what degree are you worried about the following situations? Losing my job or not finding a job | 0.00 | | 0.00 | | 0.00 | | 0.00 | | **0.43** | | No at all worried about losing my job or not finding a job | |
